# Structure-based substrate screening for an enzyme

**DOI:** 10.1186/1471-2105-10-257

**Published:** 2009-08-21

**Authors:** Tao Xu, Lujia Zhang, Xuedong Wang, Dongzhi Wei, Tianbi Li

**Affiliations:** 1 State Key Laboratory of Bioreactor Engineering, East China University of Science and Technology, Meilong Road, Shanghai, PR China; 2 Biological Chemistry Department, Rothamsted Research, Harpenden, AL5 2JQ, UK

## Abstract

**Background:**

Nowadays, more and more novel enzymes can be easily found in the whole enzyme pool with the rapid development of genetic operation. However, experimental work for substrate screening of a new enzyme is laborious, time consuming and costly. On the other hand, many computational methods have been widely used in lead screening of drug design. Seeing that the ligand-target protein system in drug design and the substrate-enzyme system in enzyme applications share the similar molecular recognition mechanism, we aim to fulfill the goal of substrate screening by in silico means in the present study.

**Results:**

A computer-aided substrate screening (CASS) system which was based on the enzyme structure was designed and employed successfully to help screen substrates of *Candida antarctica *lipase B (CALB). In this system, restricted molecular docking which was derived from the mechanism of the enzyme was applied to predict the energetically favorable poses of substrate-enzyme complexes. Thereafter, substrate conformation, distance between the oxygen atom of the alcohol part of the ester (in some compounds, this oxygen atom was replaced by nitrogen atom of the amine part of acid amine or sulfur atom of the thioester) and the hydrogen atom of imidazole of His224, distance between the carbon atom of the carbonyl group of the compound and the oxygen atom of hydroxyl group of Ser105 were used sequentially as the criteria to screen the binding poses. 223 out of 233 compounds were identified correctly for the enzyme by this screening system. Such high accuracy guaranteed the feasibility and reliability of the CASS system.

**Conclusion:**

The idea of computer-aided substrate screening is a creative combination of computational skills and enzymology. Although the case studied in this paper is tentative, high accuracy of the CASS system sheds light on the field of computer-aided substrate screening.

## Background

Enzyme catalyzes a wide variety of chemical reactions with great efficiency and specificity [1]. Applications of enzymes in industrial catalysis continue to grow because of their considerable advantages [2]. Although the classical approach of cultivating and characterizing isolates on the strain level prior to gene isolation is valid and powerful, it is severely restricted in scope [3]. So capturing the genes of organisms that have evolved as participants in biotopes promises to revolutionize and broaden enzyme applications in the chemical industry [2]. By the analysis of relationships among sequence, structure and activity [4], the function of newly obtained biocatalysts can be identified. However, broadening enzyme substrate specificity [4] is still a tough task most of the time.

On the other hand, computer-aided drug design (CADD) [5], especially different new protein inhibitors design [6-9], has been developed rapidly. Many theories and methodologies have been brought forward in this field [10-12]. More and more new drugs have been designed [13-16] by in silico methods. In view of the similar molecular recognition nature between ligand-target protein system and enzyme-substrate system [17], molecular docking [17,18] which was used in CADD to find the binding pattern between ligand and target protein was applied to broaden enzyme mapping of substrates in this study.

Although molecular docking is efficient in predicting energetically favourable poses of ligand [19], it may be inappropriate for explaining the substrate-enzyme reactions sometimes. Because in some situations, substrates may adopt energetically unfavourable poses which can not be accounted by molecular docking to facilitate the catalytic reactions that are mediated by enzymes [20]. It is especially true when the catalysis step is the actual rate-determining step. So we employed a third screening criterion in our designed screening system to address the problem (see "Distance 2 Check" section of the Method). Although the most accurate way of studying substrate-enzyme reactions is the quantum chemical (QM) level computation [21], its application in biomacromolecules is too costly to be achieved at present. So molecular docking, a rough but much less costly computational tool, was used to simulate the substrate binding step of the enzyme reaction process. Although the structure-based CASS system developed in the present study seemed simple and coarse, its screening accuracy was unexpectedly high. 223 out of 233 tested compounds were identified correctly for the enzyme by CASS. This suggests that biotechnologists can use the same computational means to reduce their mount of experimental work.

## Results and Discussion

**Figure 1:**
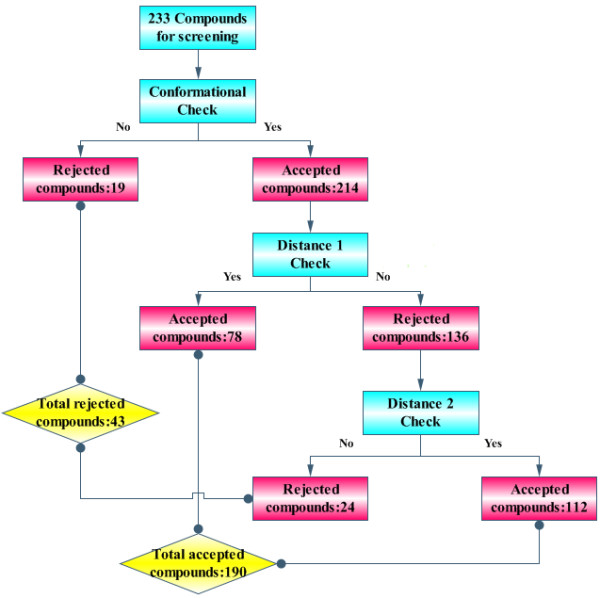
**Screening result of the CASS**.

Measurements of all binding conformations for each compound were listed in the Table S1 of Additional file 1. Then lowest energy conformations (always the first conformation) were picked out for the checking system (Table S2 of Additional file 1). As figure 1 showed, 19 out of 233 compounds were rejected by "Conformational Check". The rest 214 compounds were subject to the second screening criterion – "Distance 1 Check". Only 78 compounds were accepted. Finally, the 136 rejected compounds were screened by the third screening standard – "Distance 2 Check". Through this step, another group of 112 compounds was accepted. Altogether, 190 of the 233 compounds were accepted as potential substrates of CALB. The remnant 43 compounds were considered not to be catalyzed by the enzyme.

Generally speaking, experimental work should be carried out to inspect the accuracy of the in silico screening result. However, all the 233 compounds used in the present study had been fortunately reported by references (references were listed in Additional file 1). By comparing the virtual screening results with the reported experimental observations, we found an unexpected but inspiring result: all 190 compounds accepted by CASS system were confirmed to be catalyzed by the enzyme; 33 out of the 43 rejected compounds were verified as inappropriate substrate of the enzyme; in all, 223 out of the 233 tested compounds were identified correctly by the in silico screening system. Such high accuracy of the method (95.7%) guaranteed not only the feasibility but also the availability of the CASS system.

There were still 10 compounds which were predicted mistakenly by the CASS system. The error would probably come from molecular docking, because substrates may adopt energetically unfavourable poses which can not be accounted by molecular docking to facilitate the catalytic reactions.

## Conclusion

In the present study, the idea of computer-aided enzyme substrate screening (CASS) was introduced, designed and applied successfully to CALB. 223 out of 233 compounds were identified correctly by this in silico screening system. Such high accuracy of the method guaranteed both the feasibility and the reliability of the CASS system. Although the idea of structure-based computer-aided substrate screening sounds wonderful, its application seems more difficult than lead screening in CADD because of three main operational difficulties: (1) how to determine the 3-D structure of enzyme; (2) how to define the screening criteria to ensure availability and accuracy; (3) how to apply the screening criteria to computer software. In this light, there is still a long way to go. However, conformational and geometrical checks which were used in this study suggest clues. Our further work will revolve around the application of the CASS system to a lipase which was discovered by our own group recently [22]. We hope to broaden the substrate mapping of it with less experimental work, meanwhile we would also check again the method designed in this study.

## Methods

### Design of the computer-aided substrate screening system

**Figure 2:**
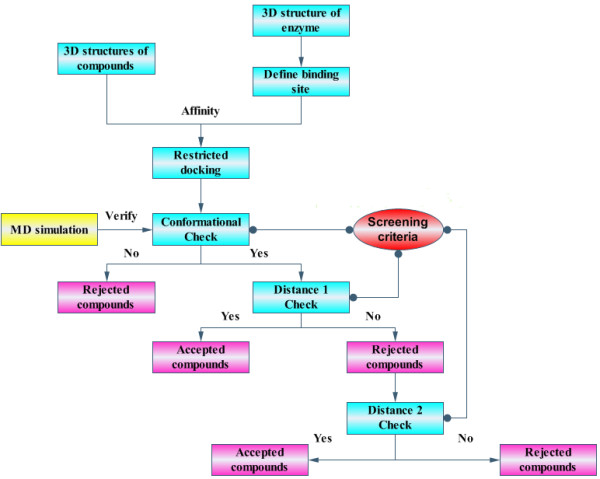
**Flow chart of the CASS**.

As figure 2 showed, tested compounds were docked into the binding site of enzyme by Affinity (InsightII, version 2000 release, Accelerys) which created at most four possible conformations between compound and enzyme. And three parameters (compound conformations, and two separate geometric distances) were measured. Then the conformation with the lowest energy was picked out and screened sequentially by three criteria: (1) "Conformational Check"; (2) "Distance 1 Check"; (3) "Distance 2 Check". All the three screening criteria, together with other details of the CASS system, would be described in the next few paragraphs in an order of what were shown in figure 2.

### Building the structures of the tested compounds

All 233 compounds which were used as tested compounds were built by Builder (InsightII, version 2000 release, Accelerys), and energy minimised by Discover (InsightII, version 2000 release, Accelerys) using the CVFF force field. Their coordinates were stored in Additional file 2.

### Structure of enzyme and binding site

CALB was studied in the present study because it had been widely used in the academic world as well as in industry as an efficient biocatalyst for asymmetric transformation of sec-alcohols and related compounds [23] due to its high activity, stability and selectivity in both aqueous and organic solutions [24].

So far there were six crystal structures of CALB in PDB databank. And the ligand free enzyme (PDB code 1TCA[25]) was used as the starting point in this study. The two N-acetyl-D-glucosamine (NAG) moieties in the structure were removed. Hydrogen atoms were added to the enzyme and water molecules. The catalytic histidine, His 224, was defined as protonated. Then an iterative series of energy minimizations were performed on the water hydrogen, enzyme hydrogen, and full water molecules. Finally, the whole system was energy minimized.

**Figure 3:**
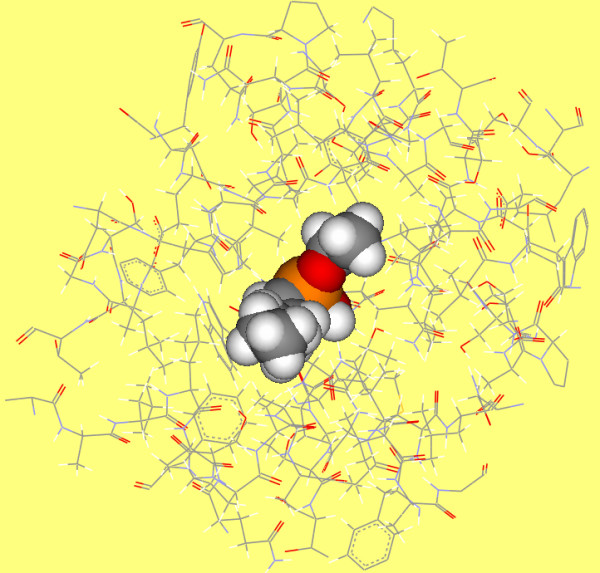
**Binding site of CALB**. The residues involved inbinding site include: 37–43, 46–48, 71–73, 76, and 79, 103–110, 131–142, 144–145, 150–159, 161, 163–164, 183, 187–193, 201–202, 223–229, 275–288. The transition state analogy – HEE in 1LBS is presented in CPK type.

The transition state analog crystal structure of CALB (PDB code 1LBS[26]) was used to help determine the binding site. Residues within 12 Å of the phosphorous atom of the N-hexylphosphonate ethyl ester (HEE) were selected (figure 3) and directly copied to the ligand free structure (1TCA) as the binding site. This seemed not well justified because ligand bound crystal structures were always used preferably for docking study in most situations. However, the reason why we copied the binding site determined by 1LBS to the ligand free structure (1TCA) was because 1TCA outperformed 1LBS in the self-docking experiment of the present study. When we docked HEE back in to 1TCA and 1LBS using the same binding site, respectively, RMSD between the docked ligand and the ligand found in crystal structure were 1.35 Å and 1.54 Å for 1TCA and 1LBS (see Additional file 3). This suggested that 1TCA could reproduce the experimentally determined ligand conformation better than 1LBS could to. Besides, energy of the binding pose of 1TCA was much lower (see Additional file 3). This indicated that using 1TCA as the target structure for docking would probably produce more stable binding pose. Finally, all atoms RMSD between free enzyme structure (1TCA) and the transition state analog crystal structure (1LBS) was only 0.4 Å. This ensured that binding site determined from 1LBS could be copied to 1TCA with little deviation.

### Docking engine – Affinity

**Figure 4:**
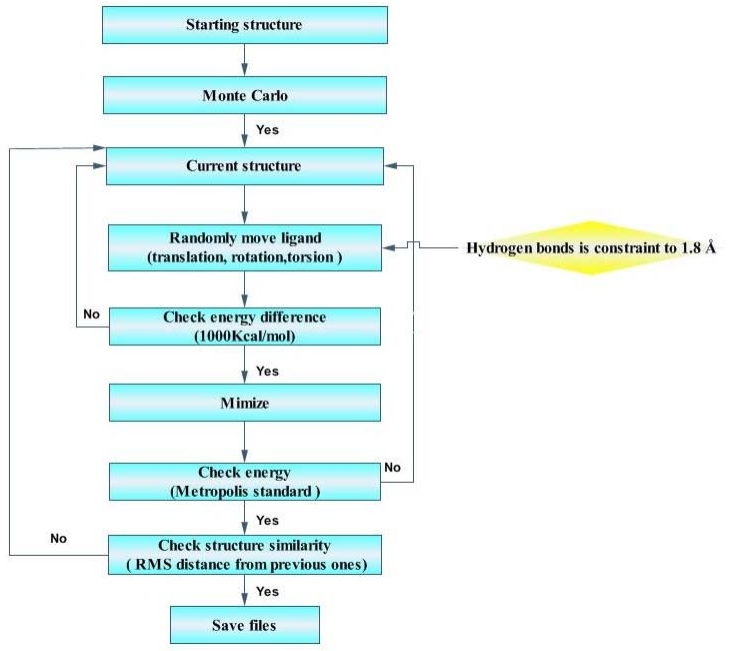
**Procedures of restricted docking by Affinity**. Main protocol of Affinity is coloured cyan. But the yellow part shows that there are three hydrogen bonds constrained at 1.8 Å.

A great deal of docking programs using different searching algorithms and scoring functions had been developed and put into practice [27-30]. In this study, an energy-driven docking method-Affinity (InsightII, version 2000 release, Accelerys) was used because it offered a very flexible and powerful docking protocol that comprised elements from Monte Carlo. Besides, Affinity adopted a full molecular mechanics force field in searching for and evaluating docked structures with both the flexibility of binding site and substrate. Figure 4 described its docking procedure. First the compounds were docked manually into the binding site of CALB, thus resulting in a roughly docked complex. Then it was energy minimized to obtain the starting structure. After that, it moved the ligand by random combination of translation, rotation, and torsional changes. The random move of ligand sampled both the orientational and conformational spaces of the ligand with respect to the receptor. It had the advantage that it could get over any energy barrier on the potential energy surface. However, randomly placing the ligand in the binding pocket in some cases could potentially lead to very severe divergences in the coulombic and vdW energies. So the scale factor for the coulombic term and vdW term is scaled down to 10^-7^. Then Affinity subsequently checked the energy of the resulting randomly moved structure. If it was within the energy tolerance parameter (1000 kcal/mol) of the previous minimized structure, it was considered to have passed the first step and the structure was then subjected to energy minimization, the second step for fine-tuning the docking. The final minimized structure was accepted or rejected based on the energy criterion and its similarity to structures found before. To prevent the search from being trapped in a local, deep potential energy well, two additional controls were adopted. Specifically, if the second energy check failed too many times (set to 4) consecutively, it suggested that the last accepted structure may be very low in energy and that it was difficult to generate new structures based on it. Thus, the current minimized structure, though it was not acceptable in energy, was used in generating new structures. Another exception was that if the search fails too many times (set to10) consecutively in finding the next acceptable structure, the program continued the search based on the current structure although it was very similar to one of the structures found previously (RMS distance being less than 0.5 Å). If the search still could not find an acceptable structure after 60 trials, the search aborted.

### Mechanism based restricted docking

**Figure 5:**
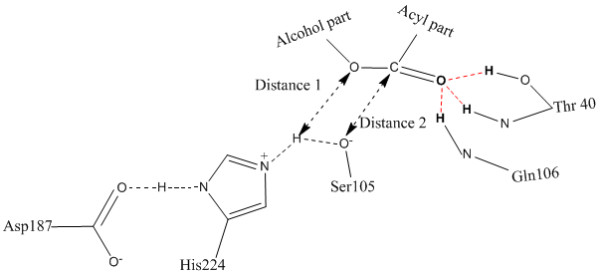
**Side-view of the active site of CALB in complex with esters**. Red dashed bonds represent the three hydrogen bonds that are kept fixed during molecular docking. And their corresponding atoms are painted bold. The hollow arrow heads show the Distance 1 and Distance 2 which are used in "Distance 1 Check" and "Distance 2 Check" of the CASS.

CALB followed the same reaction mechanism as serine hydrolase [21] and an oxyanion hole was required to stabilize the negative charge of the transition states and the acyl-enzyme intermediate during a typical reaction [31]. Essential hydrogen bonds which were involved in oxyanion hole (figure 5) were kept fixed during docking. Such hydrogen bonds fixed docking process was named "restricted docking".

### Conformational Check

X-ray diffraction of CALB indicated that its active site was made up of two pockets. One of them was for acyl part of the ester (acyl pocket) and the other for alcohol part (alcohol pocket) [26]. It seemed that the size of the acyl pocket was larger than that of alcohol pocket [31]. So we proposed a hypothetic conformational rule that the larger part of the substrate might bind into the larger binding pocket of the enzyme. And it may be used as the first screening criterion in CASS if it was proved correct.

**Figure 6:**
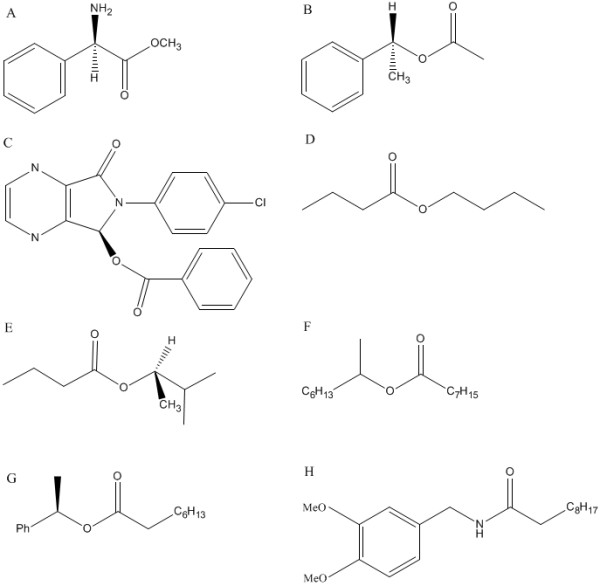
**Eight compounds for MD simulation**.

**Figure 7:**
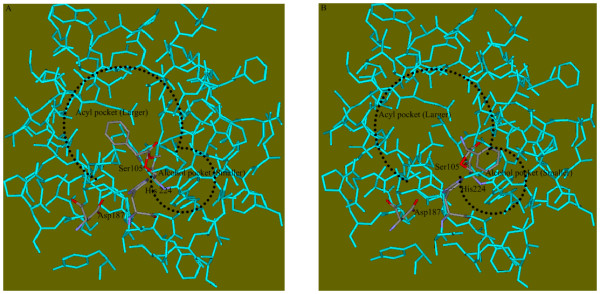
**Different initial binding conformation of transition state of the CALB-D-phenylglycinemethylester complex**. D-phenylglycinemethylester is covalently bound to Ser105. The two binding pockets are circled by dashed lines. (A) Initial binding conformation in which the larger part of the substrate binds into the larger pocket; (B) Initial binding conformation in which the larger part of the substrate binds into the smaller pocket.

MD simulations of eight enzyme-substrate transition-state complexes were carried out to inspect and verify the accuracy of the conformational rule before it was used as a screening criterion. The eight compounds (figure 6) could be classified into three groups according to the numbers of carbon atoms on each side of the ester bond (or acid amine bond for compound H). If acyl part of the compound contained more carbon atoms than the alcohol part (or amine part) did, it belonged to the "larger acyl part and smaller alcohol (or amine) part" group (compound A and H). If the alcohol part (or amine part) had more carbon atoms, it belonged to the "larger alcohol (or amine) part and smaller acyl part" group (compound B, C and E). And if both sides had the equal numbers of carbon atoms, it was called "equal size" group (compound D, F, G). For each compound, two different initial binding conformations were built as the starting structures of MD simulation (figure 7). One conformation was that the larger part of the substrate lay in the larger binding pocket (figure 7A), and the other was that the larger part of the substrate lay in the smaller binding pocket (figure 7B). The construction of each transition-state system and its following MD simulation was described in Additional file 4.

Result of the MD simulation proved the correctness of the proposed conformational rule (see table s1 of Additional file 4), so it was used as the first screening criteria of the CASS system and named "Conformational Check".

### Distance 1 Check

Once the substrate went into the binding site with correct conformation, more detailed criteria were needed to filter the substrate binding results. Two geometrically important distances were adopted in CASS. One was distance 1 which referred to distance between the oxygen atom of the alcohol part of the ester (if the substrate was an amine or thiol ester, the oxygen atom was replaced by nitrogen and sulfur atom) and the hydrogen atom of imidazole of His224 (figure 5). Distance 1 was considered as an important parameter to discriminate the enatioselectivity of secondary alcohols [32]. A shorter distance may suggest greater affinity between enzyme and substrate. So compounds which passed the "Distance 1 Check" were identified directly as the substrates of the enzyme without any further check. Compounds which failed the "Distance 1 Check" would be further checked by "Distance 2 Check".

Affinity was an energy-driven docking engine. During the docking process in this study, values of vdW potential energy were always much larger than the values of electrostatic potential energy. So the values of atom vdW radius [33,34] were used to determine the standard value of Distance 1. Distance 1 referred to distance between H atom and O (or N, S) atom in all the tested compounds. So 2.78 Å, the sum of 1.58 Å (the average vdW radius of the O, N, S) and 1.2 Å (the vdW radius of H), was used as the standard value of "Distance 1 Check".

### Distance 2 Check

In some cases, Affinity may find no energy favorably binding conformations and would just give the energy unfavorably binding conformations. This was allowable in our substrate screening system. Because some compounds would take the energy unfavorable binding patterns to facilitate the reaction of compounds with biocatalyst. To address the problem, "Distance 2 Check" was adopted. It referred to distance between the carbon atom of the carbonyl group of the candidate compound and the oxygen atom of hydroxyl group of Ser105 (figure 5). And it was a subsidiary screening criterion of" Distance 1 Check" to guarantee the sensitivity and availability of the in silico system. A shorter distance was believed to better facilitate the nucleophilic attack of Ser105 to the carbonyl group of compounds. Only compounds which failed the "Distance 1 Check" could be further subjected to the "Distance 2 Check". 3.12 Å (sum of the atom vdW radius of C and O) was used as the standard value, because "Distance 2 Check" contains C and O atom in all compounds.

## List of abbreviations

CALB: *Candida antarctica *lipase B; CADD: Computer-aided drug design; CASS: Computer-aided substrate screening; QM: Quantum chemical; MD: Molecular dynamics; vdW: Van der Waals; HEE: N-hexylphosphonate ethyl ester; RMSD: Root mean square deviation.

## Authors' contributions

TX and LZ constructed the idea of CASS. TX did the computational work and the following analysis. All authors were participated in the drafting the manuscript and approved the final version.

## Supplementary Material

Additional file 1 **Screening results**. Table S1 displays the all binding conformations of each compound. Table S2 shows the screening result of CASS system. Besides, all the references about tested compounds are listed. Click here for file

Additional file 2 **3D structures of all compounds used for substrate screening**. All structures are stored in ".car" format (Insight-II readable). Click here for file

Additional file 3 **Docking N-hexylphosphonate ethyl ester (HEE) in **1TCA** and **1LBS. This part compares the results of the self-docking experiment using 1TCA and 1LBS as the target structure in detail. Click here for file

Additional file 4 **MD simulation of the transition state of CALB-substrate complex**. This part displays a detailed description of the preparation of transition state of enzyme-substrate complex and the following MD simulation results. Table S1 shows the result of MD simulation. Click here for file
