# Supplementary material for: Structure-based substrate screening for an enzyme
Source: BMC Bioinformatics. 2009 Aug 21;10:257. doi: 10.1186/1471-2105-10-257 (PMC2745390; doi:10.1186/1471-2105-10-257)
Supplement: Additional file 1 — Screening results. Table S1 displays the all binding conformations of each compound. Table S2 shows the screening result of CASS system. Besides, all the references about tested compounds are listed. [file 1471-2105-10-257-S1.doc]

**Binding Postures of Each Compound**

In the present study, *Affinity* created at most four binding postures between enzyme-substrate. These postures were listed in an order of increasing energy (Table S1). In some cases, *Affinity* could not find four postures, so the notation of “／” was used for these postures. Besides, “other conformation” represented strange binding conformations, for example: acyl part (or alcohol) of the ester bound into neither binding pockets. Distance 1 and distance 2 were shown in figure 5.

Table S1 Binding postures between enzyme and all 233 compounds

| Compounds | Number of postures | Energy (kJ/mol) | Conformation check | Distance 1(Å) | Distance 2(Å) |
| --- | --- | --- | --- | --- | --- |
| 1 | 1 | 98.262 | pass | 3.28 | 2.23 |
|  | 2 | 98.263 | pass | 3.21 | 3.01 |
|  | 3 | 98.27 | pass | 4.49 | 2.91 |
|  | 4 | 98.316 | pass | 4.76 | 2.89 |
|  |  |  |  |  |  |
| 2 | 1 | 98.24 | pass | 6.39 | 4.68 |
|  | 2 | 98.241 | pass | 2.49 | 2.33 |
|  | 3 | 98.241 | pass | 2.19 | 2.82 |
|  | 4 | 98.253 | fail | 4.79 | 3.14 |
|  |  |  |  |  |  |
| 3 | 1 | 98.061 | pass | 2.83 | 2.51 |
|  | 2 | 98.08 | pass | 3.52 | 2 |
|  | 3 | 97.095 | pass | 4.03 | 2.8 |
|  | 4 | 98.107 | pass | 3.04 | 2.99 |
|  |  |  |  |  |  |
| 4 | 1 | 98.122 | pass | 3.74 | 2.29 |
|  | 2 | 98.13 | pass | 2.59 | 2.77 |
|  | 3 | 98.163 | pass | 4.84 | 2.29 |
|  | 4 | 98.181 | pass | 4.97 | 3.46 |
|  |  |  |  |  |  |
| 5 | 1 | 98.137 | pass | 2.66 | 2.09 |
|  | 2 | 98.146 | pass | 3.57 | 2.37 |
|  | 3 | 98.154 | pass | 4.98 | 3.25 |
|  | 4 | 98.158 | pass | 3.5 | 2.27 |
|  |  |  |  |  |  |
| 6 | 1 | 98.144 | fail | 3.06 | 2.53 |
|  | 2 | 98.146 | fail | 1.67 | 1.87 |
|  | 3 | 98.146 | fail | 4.14 | 2.56 |
|  | 4 | 98.156 | fail | 2.4 | 1.96 |
|  |  |  |  |  |  |
| 7 | 1 | 98.103 | pass | 2.5 | 2.32 |
|  | 2 | 98.195 | pass | 2.99 | 2.28 |
|  | 3 | 98.103 | pass | 4.32 | 2.59 |
|  | 4 | 98.203 | pass | 2.78 | 1.93 |
|  |  |  |  |  |  |
| 8 | 1 | 98.122 | pass | 4.29 | 2.86 |
|  | 2 | 98.187 | pass | 3.77 | 2.57 |
|  | 3 | 98.209 | pass | 3.13 | 1.72 |
|  | 4 | 98.217 | fail | 4.49 | 2.48 |
|  |  |  |  |  |  |
| 9 | 1 | 98.023 | pass | 3.85 | 2.65 |
|  | 2 | 98.036 | pass | 3.98 | 2.44 |
|  | 3 | ／ | ／ | ／ | ／ |
|  | 4 | ／ | ／ | ／ | ／ |
|  |  |  |  |  |  |
| 10 | 1 | 97.984 | pass | 5.12 | 2.59 |
|  | 2 | 97.984 | pass | 4.22 | 2.65 |
|  | 3 | 98.024 | fail | 4.32 | 2.56 |
|  | 4 | 98.029 | pass | 3.34 | 1.46 |
|  |  |  |  |  |  |
| 11 | 1 | 98.341 | pass | 2.76 | 2.14 |
|  | 2 | 98.314 | pass | 2.4 | 2.04 |
|  | 3 | 98.394 | pass | 3.47 | 2.5 |
|  | 4 | 98.394 | pass | 3.76 | 1.91 |
|  |  |  |  |  |  |
| 12 | 1 | 98.382 | pass | 3.74 | 3.24 |
|  | 2 | 98.382 | fail | 3.75 | 1.86 |
|  | 3 | 98.419 | pass | 2.67 | 1.8 |
|  | 4 | ／ | ／ | ／ | ／ |
|  |  |  |  |  |  |
| 13 | 1 | 97.867 | pass | 2.37 | 1.69 |
|  | 2 | 97.869 | pass | 4.8 | 2.47 |
|  | 3 | 97.87 | pass | 5.44 | 3.24 |
|  | 4 | 97.873 | fail | 2.53 | 2.08 |
|  |  |  |  |  |  |
| 14 | 1 | 97.788 | pass | 4.2 | 3.53 |
|  | 2 | 97.798 | pass | 3.03 | 2.3 |
|  | 3 | 97.799 | fail | 2.46 | 2.11 |
|  | 4 | 97.8 | pass | 2.77 | 2.45 |
|  |  |  |  |  |  |
| 15 | 1 | 97.943 | pass | 2.2 | 1.94 |
|  | 2 | 97.955 | fail | 2.1 | 2.35 |
|  | 3 | 97.987 | pass | 3.63 | 1.94 |
|  | 4 | 98.001 | pass | 3.83 | 2.63 |
|  |  |  |  |  |  |
| 16 | 1 | 97.988 | fail | 3.07 | 2.05 |
|  | 2 | 98.025 | fail | 4.29 | 2.63 |
|  | 3 | ／ | ／ | ／ | ／ |
|  | 4 | ／ | ／ | ／ | ／ |
|  |  |  |  |  |  |
| 17 | 1 | 97.868 | pass | 3.06 | 3.86 |
|  | 2 | 97.879 | other conforamtion | | |
|  | 3 | 97.88 | pass | 3.83 | 3.14 |
|  | 4 | 97.888 | other conforamtion | | |
|  |  |  |  |  |  |
| 18 | 1 | 103.24 | pass | 1.98 | 2.81 |
|  | 2 | 103.2 | pass | 3.86 | 3.25 |
|  | 3 | 103.25 | pass | 3.06 | 2.5 |
|  | 4 | 103.25 | other conforamtion | | |
|  |  |  |  |  |  |
| 19 | 1 | 103.14 | pass | 3.04 | 2.8 |
|  | 2 | 103.14 | other conforamtion | | |
|  | 3 | 103.15 | pass | 3.4 | 3.11 |
|  | 4 | 103.16 | pass | 3.17 | 2.95 |
|  |  |  |  |  |  |
| 20 | 1 | 103.22 | pass | 3.41 | 1.86 |
|  | 2 | 103.24 | other conforamtion | | |
|  | 3 | 103.26 | other conforamtion | | |
|  | 4 | 103.27 | other conforamtion | | |
|  |  |  |  |  |  |
| 21 | 1 | 103.2 | pass | 2.75 | 1.81 |
|  | 2 | 103.21 | pass | 3.74 | 2.77 |
|  | 3 | 103.22 | fail | 3.68 | 1.84 |
|  | 4 | ／ | ／ | ／ | ／ |
|  |  |  |  |  |  |
| 22 | 1 | 103.37 | pass | 2.72 | 1.22 |
|  | 2 | 103.42 | pass | 4.87 | 3.56 |
|  | 3 | ／ | ／ | ／ | ／ |
|  | 4 | ／ | ／ | ／ | ／ |
|  |  |  |  |  |  |
| 23 | 1 | 103.32 | other conforamtion | | |
|  | 2 | 103.32 | other conforamtion | | |
|  | 3 | 103.36 | pass | 4.44 | 2.54 |
|  | 4 | ／ | ／ | ／ | ／ |
|  |  |  |  |  |  |
| 24 | 1 | 103.31 | pass | 1.34 | 2.56 |
|  | 2 | 103.32 | pass | 2.82 | 3.63 |
|  | 3 | 103.35 | pass | 3.25 | 3.73 |
|  | 4 | 103.39 | other conforamtion | | |
|  |  |  |  |  |  |
| 25 | 1 | 105.91 | fail | 4.32 | 2.28 |
|  | 2 | 105.93 | fail | 3.97 | 2.77 |
|  | 3 | 105.93 | fail | 3.28 | 2.28 |
|  | 4 | 106 | other conforamtion | | |
|  |  |  |  |  |  |
| 26 | 1 | 105.94 | pass | 2.73 | 2.08 |
|  | 2 | 105.94 | fail | 4.49 | 2.62 |
|  | 3 | 105.95 | other conforamtion | | |
|  | 4 | 105.95 | other conforamtion | | |
|  |  |  |  |  |  |
| 27 | 1 | 105.85 | fail | 2.02 | 2.31 |
|  | 2 | 105.86 | fail | 4.05 | 3.15 |
|  | 3 | 105.87 | fail | 2.02 | 2.31 |
|  | 4 | 105.87 | fail | 2.72 | 2.92 |
|  |  |  |  |  |  |
| 28 | 1 | 105.87 | pass | 2.89 | 2.15 |
|  | 2 | 105.86 | pass | 3.83 | 2.61 |
|  | 3 | 105.88 | pass | 3.15 | 1.57 |
|  | 4 | 105.88 | other conforamtion | | |
|  |  |  |  |  |  |
| 29 | 1 | 98.033 | pass | 5.54 | 3.08 |
|  | 2 | 98.058 | other conforamtion | | |
|  | 3 | 98.078 | other conforamtion | | |
|  | 4 | 98.083 | other conforamtion | | |
|  |  |  |  |  |  |
| 30 | 1 | 98.006 | pass | 3.65 | 3.03 |
|  | 2 | 98.101 | other conforamtion | | |
|  | 3 | 98.131 | other conforamtion | | |
|  | 4 | ／ | ／ | ／ | ／ |
|  |  |  |  |  |  |
| 31 | 1 | 97.953 | pass | 2.98 | 3.15 |
|  | 2 | 97.948 | other conforamtion | | |
|  | 3 | ／ | ／ | ／ | ／ |
|  | 4 | ／ | ／ | ／ | ／ |
|  |  |  |  |  |  |
| 32 | 1 | 97.767 | pass | 3.69 | 2.59 |
|  | 2 | 97.837 | other conforamtion | | |
|  | 3 | 97.896 | pass | 4.17 | 3.09 |
|  | 4 | 97.984 | pass | 4.24 | 2.78 |
|  |  |  |  |  |  |
| 33 | 1 | 99.202 | pass | 3.51 | 3.41 |
|  | 2 | 99.208 | pass | 3.8 | 1.82 |
|  | 3 | 99.211 | pass | 3.95 | 2.49 |
|  | 4 | 99.213 | pass | 3.73 | 1.97 |
|  |  |  |  |  |  |
| 34 | 1 | 99.175 | other conforamtion | | |
|  | 2 | 99.214 | other conforamtion | | |
|  | 3 | 99.216 | pass | 2.57 | 1.99 |
|  | 4 | 99.217 | pass | 4.01 | 1.8 |
|  |  |  |  |  |  |
| 35 | 1 | 97.996 | pass | 2.36 | 1.72 |
|  | 2 | ／ | ／ | ／ | ／ |
|  | 3 | ／ | ／ | ／ | ／ |
|  | 4 | ／ | ／ | ／ | ／ |
|  |  |  |  |  |  |
| 36 | 1 | 98.171 | pass | 3.5 | 2.13 |
|  | 2 | 98.043 | pass | 2.77 | 2.22 |
|  | 3 | ／ | ／ | ／ | ／ |
|  | 4 | ／ | ／ | ／ | ／ |
|  |  |  |  |  |  |
| 37 | 1 | 98.145 | other conforamtion | | |
|  | 2 | 98.174 | other conforamtion | | |
|  | 3 | 98.212 | pass | 4.3 | 3.17 |
|  | 4 | ／ | ／ | ／ | ／ |
|  |  |  |  |  |  |
| 38 | 1 | 98.147 | pass | 3.69 | 2.14 |
|  | 2 | 98.153 | pass | 4.23 | 1.86 |
|  | 3 | 98.262 | other conforamtion | | |
|  | 4 | 98.305 | pass | 3.45 | 2.53 |
|  |  |  |  |  |  |
| 39 | 1 | 98.277 | fail | 3.08 | 2.21 |
|  | 2 | 98.329 | fail | 2.45 | 2.09 |
|  | 3 | 98.392 | other conforamtion | | |
|  | 4 | 98.426 | other conforamtion | | |
|  |  |  |  |  |  |
| 40 | 1 | 98.215 | fail | 3.99 | 2.03 |
|  | 2 | 98.264 | other conforamtion | | |
|  | 3 | 98.344 | other conforamtion | | |
|  | 4 | 98.511 | other conforamtion | | |
|  |  |  |  |  |  |
| 41 | 1 | 98.399 | fail | 2.66 | 1.72 |
|  | 2 | 98.393 | fail | 2.71 | 1.56 |
|  | 3 | ／ | ／ | ／ | ／ |
|  | 4 | ／ | ／ | ／ | ／ |
|  |  |  |  |  |  |
| 42 | 1 | 98.34 | pass | 2.9 | 3.35 |
|  | 2 | 98.348 | pass | 2.28 | 2.36 |
|  | 3 | 98.352 | other conforamtion | | |
|  | 4 | 98.364 | other conforamtion | | |
|  |  |  |  |  |  |
| 43 | 1 | 119.24 | pass | 2.94 | 2.37 |
|  | 2 | 119.24 | pass | 4.19 | 3.19 |
|  | 3 | 119.8 | other conforamtion | | |
|  | 4 | 120.02 | other conforamtion | | |
|  |  |  |  |  |  |
| 44 | 1 | 119.13 | pass | 1.29 | 2.49 |
|  | 2 | 119.14 | other conforamtion | | |
|  | 3 | 119.14 | fail | 5.81 | 3.15 |
|  | 4 | 119.15 | other conforamtion | | |
|  |  |  |  |  |  |
| 45 | 1 | 116.09 | pass | 4.93 | 3.05 |
|  | 2 | ／ | ／ | ／ | ／ |
|  | 3 | ／ | ／ | ／ | ／ |
|  | 4 | ／ | ／ | ／ | ／ |
|  |  |  |  |  |  |
| 46 | 1 | 115.89 | pass | 3.13 | 2.95 |
|  | 2 | ／ | ／ | ／ | ／ |
|  | 3 | ／ | ／ | ／ | ／ |
|  | 4 | ／ | ／ | ／ | ／ |
|  |  |  |  |  |  |
| 47 | 1 | 97.917 | pass | 3.09 | 1.64 |
|  | 2 | 97.927 | pass | 4.36 | 2.08 |
|  | 3 | 97.95 | fail | 3.19 | 1.46 |
|  | 4 | 97.974 | fail | 4.22 | 1.46 |
|  |  |  |  |  |  |
| 48 | 1 | 97.979 | pass | 5.2 | 3.01 |
|  | 2 | 97.998 | fail | 4.08 | 2.64 |
|  | 3 | 98.052 | fail | 4.09 | 2.89 |
|  | 4 | ／ | ／ | ／ | ／ |
|  |  |  |  |  |  |
| 49 | 1 | 97.942 | pass | 3.16 | 2.08 |
|  | 2 | 97.95 | other conforamtion | | |
|  | 3 | 97.953 | other conforamtion | | |
|  | 4 | 97.963 | pass | 5.02 | 3.28 |
|  |  |  |  |  |  |
| 50 | 1 | 97.96 | pass | 4.57 | 2.61 |
|  | 2 | 97.956 | fail | 3.17 | 1.8 |
|  | 3 | 97.96 | pass | 5.02 | 2.98 |
|  | 4 | 98.035 | fail | 3.2 | 2.41 |
|  |  |  |  |  |  |
| 51 | 1 | 97.911 | pass | 2.75 | 1.62 |
|  | 2 | 97.929 | pass | 4.22 | 3.51 |
|  | 3 | 97.967 | pass | 4.02 | 3.3 |
|  | 4 | 97.991 | other conforamtion | | |
|  |  |  |  |  |  |
| 52 | 1 | 97.802 | pass | 3.24 | 2.88 |
|  | 2 | 97.802 | fail | 3.74 | 2.98 |
|  | 3 | 97.803 | fail | 4.12 | 2.96 |
|  | 4 | 87.81 | fail | 3.79 | 2.61 |
|  |  |  |  |  |  |
| 53 | 1 | 98.041 | pass | 2.17 | 2.23 |
|  | 2 | 98.041 | pass | 4.89 | 2.72 |
|  | 3 | 98.042 | pass | 4.91 | 2.73 |
|  | 4 | 98.043 | fail | 2.69 | 2 |
|  |  |  |  |  |  |
| 54 | 1 | 97.915 | pass | 2.47 | 2.02 |
|  | 2 | 97.916 | fail | 3.32 | 2.26 |
|  | 3 | 97.917 | pass | 5.03 | 2.26 |
|  | 4 | 97.918 | fail | 4.77 | 2.4 |
|  |  |  |  |  |  |
| 55 | 1 | 98.037 | pass | 2.72 | 2.26 |
|  | 2 | 98.028 | pass | 4.68 | 1.89 |
|  | 3 | 98.028 | pass | 5.42 | 2.84 |
|  | 4 | 98.03 | fail | 3.38 | 2.17 |
|  |  |  |  |  |  |
| 56 | 1 | 98.011 | other conforamtion | | |
|  | 2 | 98.049 | pass | 2.39 | 2.94 |
|  | 3 | 98.059 | pass | 3.38 | 1.71 |
|  | 4 | 98.467 | pass | 5.89 | 2.8 |
|  |  |  |  |  |  |
| 57 | 1 | 97.863 | pass | 2.28 | 1.83 |
|  | 2 | 97.867 | pass | 2.45 | 2.08 |
|  | 3 | 97.87 | fail | 3.9 | 2.36 |
|  | 4 | 97.876 | fail | 3.46 | 2.43 |
|  |  |  |  |  |  |
| 58 | 1 | 97.82 | pass | 4.48 | 2.99 |
|  | 2 | 97.821 | fail | 4.29 | 2.69 |
|  | 3 | 97.825 | pass | 4.43 | 3.06 |
|  | 4 | 97.835 | fail | 4.09 | 2.71 |
|  |  |  |  |  |  |
| 59 | 1 | 97.836 | pass | 2.55 | 3.25 |
|  | 2 | 97.847 | fail | 3.94 | 2.94 |
|  | 3 | 97.858 | fail | 3.23 | 2.67 |
|  | 4 | 97.881 | fail | 4.97 | 2.42 |
|  |  |  |  |  |  |
| 60 | 1 | 98.065 | pass | 3.97 | 2.78 |
|  | 2 | 98.066 | fail | 4.11 | 1.96 |
|  | 3 | 98.069 | fail | 4.9 | 2.46 |
|  | 4 | 98.075 | other conforamtion | | |
|  |  |  |  |  |  |
| 61 | 1 | 97.926 | pass | 3.22 | 2.75 |
|  | 2 | 97.927 | pass | 4.15 | 2.61 |
|  | 3 | 97.948 | fail | 6.16 | 1.87 |
|  | 4 | 97.953 | pass | 3.96 | 3.03 |
|  |  |  |  |  |  |
| 62 | 1 | 99.204 | pass | 2.89 | 2.29 |
|  | 2 | 99.205 | pass | 4.8 | 2.64 |
|  | 3 | 99.207 | pass | 4.64 | 2.27 |
|  | 4 | 99.207 | fail | 3.43 | 2.73 |
|  |  |  |  |  |  |
| 63 | 1 | 104.31 | pass | 3.69 | 2.31 |
|  | 2 | 104.48 | fail | 3.59 | 2.57 |
|  | 3 | 104.51 | fail | 4.78 | 2.56 |
|  | 4 | ／ | ／ | ／ | ／ |
|  |  |  |  |  |  |
| 64 | 1 | 98.863 | pass | 2.08 | 1.97 |
|  | 2 | 98.864 | pass | 3.84 | 1.78 |
|  | 3 | 98.864 | pass | 4.03 | 1.97 |
|  | 4 | 98.867 | fail | 4.19 | 2.45 |
|  |  |  |  |  |  |
| 65 | 1 | 98.685 | pass | 4.42 | 2.98 |
|  | 2 | 98.69 | pass | 5.05 | 2.98 |
|  | 3 | 98.695 | fail | 5.04 | 2.58 |
|  | 4 | ／ | ／ | ／ | ／ |
|  |  |  |  |  |  |
| 66 | 1 | 101.07 | pass | 2.3 | 2.17 |
|  | 2 | 101.08 | fail | 3 | 2.43 |
|  | 3 | 101.08 | fail | 2.91 | 2.63 |
|  | 4 | 101.08 | pass | 3.86 | 1.39 |
|  |  |  |  |  |  |
| 67 | 1 | 100.4 | pass | 2.33 | 1.52 |
|  | 2 | 100.41 | pass | 5.16 | 2.38 |
|  | 3 | 100.41 | pass | 3.98 | 2.04 |
|  | 4 | 100.41 | pass | 5.46 | 2.95 |
|  |  |  |  |  |  |
| 68 | 1 | 98.629 | pass | 3.03 | 2.73 |
|  | 2 | 98.634 | pass | 4.62 | 2.56 |
|  | 3 | 98.634 | fail | 3.56 | 3.17 |
|  | 4 | 98.634 | fail | 3.01 | 2.93 |
|  |  |  |  |  |  |
| 69 | 1 | 109.03 | pass | 2.43 | 2.97 |
|  | 2 | 109.03 | pass | 2.53 | 2.49 |
|  | 3 | 109.04 | pass | 2.72 | 2.64 |
|  | 4 | 109.04 | pass | 3.11 | 3.18 |
|  |  |  |  |  |  |
| 70 | 1 | 102.18 | pass | 2.33 | 2.52 |
|  | 2 | 102.21 | pass | 3.91 | 2.57 |
|  | 3 | 102.23 | pass | 4.01 | 2.98 |
|  | 4 | 102.23 | pass | 5.64 | 2.64 |
|  |  |  |  |  |  |
| 71 | 1 | 118.98 | pass | 3.47 | 2.73 |
|  | 2 | 118.98 | pass | 3.62 | 1.99 |
|  | 3 | 118.99 | pass | 3.67 | 2.47 |
|  | 4 | 118.99 | fail | 5.02 | 2.35 |
|  |  |  |  |  |  |
| 72 | 1 | 99.247 | pass | 1.96 | 2.15 |
|  | 2 | 99.253 | pass | 2.81 | 1.99 |
|  | 3 | 99.267 | other conforamtion | | |
|  | 4 | 99.273 | pass | 3.31 | 1.94 |
|  |  |  |  |  |  |
| 73 | 1 | 97.818 | pass | 2.2 | 1.56 |
|  | 2 | 97.823 | pass | 2.82 | 2.51 |
|  | 3 | 97.826 | fail | 5.02 | 3.32 |
|  | 4 | 97.828 | pass | 2.81 | 1.51 |
|  |  |  |  |  |  |
| 74 | 1 | 98.323 | pass | 3.77 | 2.1 |
|  | 2 | 98.324 | pass | 4.07 | 3.01 |
|  | 3 | 98.331 | pass | 3.89 | 3.09 |
|  | 4 | 98.355 | pass | 4.26 | 3.15 |
|  |  |  |  |  |  |
| 75 | 1 | 98.473 | pass | 3.63 | 2.06 |
|  | 2 | 98.479 | fail | 4.44 | 3.99 |
|  | 3 | 98.482 | fail | 4.06 | 2.47 |
|  | 4 | 98.514 | fail | 5.32 | 3.43 |
|  |  |  |  |  |  |
| 76 | 1 | 101.21 | pass | 3.83 | 2.27 |
|  | 2 | 101.39 | pass | 4.62 | 2.53 |
|  | 3 | 101.4 | pass | 4.97 | 2.44 |
|  | 4 | 101.4 | pass | 3.94 | 2.17 |
|  |  |  |  |  |  |
| 77 | 1 | 102.59 | pass | 2.53 | 2.08 |
|  | 2 | 102.69 | pass | 4 | 2.99 |
|  | 3 | 102.69 | pass | 4.81 | 2.64 |
|  | 4 | 102.69 | pass | 4.72 | 2.8 |
|  |  |  |  |  |  |
| 78 | 1 | 104.07 | pass | 2.69 | 1.74 |
|  | 2 | 104.1 | fail | 4.93 | 2.58 |
|  | 3 | 104.1 | pass | 4.44 | 2.56 |
|  | 4 | 104.11 | fail | 3.61 | 2.43 |
|  |  |  |  |  |  |
| 79 | 1 | 98.172 | pass | 4.2 | 2.59 |
|  | 2 | 98.315 | fail | 4.01 | 1.96 |
|  | 3 | ／ | ／ | ／ | ／ |
|  | 4 | ／ | ／ | ／ | ／ |
|  |  |  |  |  |  |
| 80 | 1 | 98.167 | pass | 2.08 | 1.4 |
|  | 2 | 98.178 | fail | 2.08 | 1.41 |
|  | 3 | 98.235 | pass | 3.11 | 1.6 |
|  | 4 | 98.26 | fail | 4.11 | 2.17 |
|  |  |  |  |  |  |
| 81 | 1 | 98.181 | pass | 3.21 | 1.64 |
|  | 2 | 98.12 | pass | 3.35 | 2.08 |
|  | 3 | 98.2 | pass | 3.09 | 3.09 |
|  | 4 | 98.2 | pass | 2.82 | 2.4 |
|  |  |  |  |  |  |
| 82 | 1 | 98.199 | pass | 2.03 | 2.2 |
|  | 2 | 98.256 | pass | 4.76 | 2.85 |
|  | 3 | ／ | ／ | ／ | ／ |
|  | 4 | ／ | ／ | ／ | ／ |
|  |  |  |  |  |  |
| 83 | 1 | 98.131 | pass | 3.19 | 2.76 |
|  | 2 | 98.156 | pass | 4.95 | 2.71 |
|  | 3 | 98.161 | pass | 4.16 | 3.04 |
|  | 4 | 98.165 | pass | 3.26 | 1.53 |
|  |  |  |  |  |  |
| 84 | 1 | 97.898 | pass | 4.15 | 2.34 |
|  | 2 | 97.927 | pass | 4.36 | 2.38 |
|  | 3 | 97.948 | fail | 4.93 | 1.55 |
|  | 4 | 97.981 | fail | 3.52 | 2.47 |
|  |  |  |  |  |  |
| 85 | 1 | 97.906 | pass | 4.09 | 1.92 |
|  | 2 | 97.909 | fail | 4.31 | 2.99 |
|  | 3 | 97.966 | pass | 4.84 | 2.26 |
|  | 4 | 97.972 | fail | 4.89 | 2.15 |
|  |  |  |  |  |  |
| 86 | 1 | 97.907 | pass | 3.56 | 2.13 |
|  | 2 | ／ | ／ | ／ | ／ |
|  | 3 | ／ | ／ | ／ | ／ |
|  | 4 | ／ | ／ | ／ | ／ |
|  |  |  |  |  |  |
| 87 | 1 | 97.901 | pass | 2.59 | 2.05 |
|  | 2 | 97.906 | pass | 5.51 | 2.59 |
|  | 3 | 97.911 | pass | 5.68 | 3.02 |
|  | 4 | 97.97 | pass | 5.71 | 2.85 |
|  |  |  |  |  |  |
| 88 | 1 | 97.966 | pass | 3.2 | 1.98 |
|  | 2 | 97.986 | fail | 5.6 | 2.93 |
|  | 3 | 97.998 | fail | 5.12 | 2.32 |
|  | 4 | ／ | ／ | ／ | ／ |
|  |  |  |  |  |  |
| 89 | 1 | 98.002 | pass | 3.38 | 2.5 |
|  | 2 | 98.006 | fail | 5.22 | 3.53 |
|  | 3 | 98.02 | fail | 4.2 | 3.15 |
|  | 4 | 98.042 | other conforamtion | | |
|  |  |  |  |  |  |
| 90 | 1 | 97.879 | pass | 2.22 | 1.8 |
|  | 2 | 97.897 | other conforamtion | | |
|  | 3 | 97.91 | fail | 3.36 | 2.49 |
|  | 4 | 97.931 | pass | 3.83 | 3.13 |
|  |  |  |  |  |  |
| 91 | 1 | 97.874 | pass | 2.99 | 2.34 |
|  | 2 | 97.895 | pass | 4.49 | 3.52 |
|  | 3 | 97.911 | pass | 3.45 | 2.63 |
|  | 4 | 97.963 | pass | 5.17 | 2.37 |
|  |  |  |  |  |  |
| 92 | 1 | 97.972 | pass | 3.78 | 2.78 |
|  | 2 | 97.972 | pass | 4.05 | 2.83 |
|  | 3 | 97.976 | pass | 4.52 | 3.32 |
|  | 4 | 98.001 | pass | 3.94 | 2.41 |
|  |  |  |  |  |  |
| 93 | 1 | 97.926 | pass | 3.78 | 1.88 |
|  | 2 | 98.025 | fail | 6 | 3.14 |
|  | 3 | ／ | ／ | ／ | ／ |
|  | 4 | ／ | ／ | ／ | ／ |
|  |  |  |  |  |  |
| 94 | 1 | 97.934 | pass | 2.72 | 2.49 |
|  | 2 | 97.935 | fail | 4.15 | 1.79 |
|  | 3 | 97.952 | pass | 4.71 | 3.16 |
|  | 4 | 97.957 | pass | 3.2 | 2.29 |
|  |  |  |  |  |  |
| 95 | 1 | 99.205 | pass | 1.69 | 2.43 |
|  | 2 | 99.251 | pass | 5.57 | 2.63 |
|  | 3 | 99.253 | pass | 3.57 | 3.48 |
|  | 4 | 99.288 | pass | 3.52 | 1.45 |
|  |  |  |  |  |  |
| 96 | 1 | 97.923 | pass | 3.04 | 2.16 |
|  | 2 | 97.932 | fail | 5.51 | 2.85 |
|  | 3 | ／ | ／ | ／ | ／ |
|  | 4 | ／ | ／ | ／ | ／ |
|  |  |  |  |  |  |
| 97 | 1 | 98.13 | pass | 3.49 | 1.95 |
|  | 2 | 98.166 | pass | 4.64 | 3.11 |
|  | 3 | 98.195 | pass | 6.9 | 4.77 |
|  | 4 | 98.407 | pass | 6.03 | 3.69 |
|  |  |  |  |  |  |
| 98 | 1 | 97.983 | pass | 4.07 | 2.67 |
|  | 2 | 97.983 | fail | 4.92 | 3.63 |
|  | 3 | 97.986 | fail | 4.43 | 3.02 |
|  | 4 | 97.989 | fail | 5.37 | 3.58 |
|  |  |  |  |  |  |
| 99 | 1 | 98.269 | pass | 2.64 | 2.02 |
|  | 2 | 98.271 | pass | 4.42 | 2.79 |
|  | 3 | 98.347 | other conforamtion | | |
|  | 4 | 98.403 | fail | 3.71 | 1.87 |
|  |  |  |  |  |  |
| 100 | 1 | 97.919 | pass | 2.37 | 2.25 |
|  | 2 | 97.928 | fail | 2.59 | 2.25 |
|  | 3 | 97.934 | fail | 3.2 | 2.27 |
|  | 4 | 97.939 | pass | 4.47 | 2.3 |
|  |  |  |  |  |  |
| 101 | 1 | 97.922 | pass | 2.06 | 2.15 |
|  | 2 | 97.963 | fail | 5.77 | 3.13 |
|  | 3 | ／ | ／ | ／ | ／ |
|  | 4 | ／ | ／ | ／ | ／ |
|  |  |  |  |  |  |
| 102 | 1 | 98.169 | pass | 3.01 | 2.26 |
|  | 2 | 98.173 | fail | 4.12 | 2.53 |
|  | 3 | ／ | ／ | ／ | ／ |
|  | 4 | ／ | ／ | ／ | ／ |
|  |  |  |  |  |  |
| 103 | 1 | 97.873 | pass | 2.41 | 1.9 |
|  | 2 | 97.874 | fail | 6.15 | 3.45 |
|  | 3 | 97.876 | fail | 3.37 | 2.46 |
|  | 4 | 97.876 | fail | 3.96 | 2.44 |
|  |  |  |  |  |  |
| 104 | 1 | 99.301 | pass | 1.88 | 1.79 |
|  | 2 | 99.303 | pass | 4.14 | 2.58 |
|  | 3 | 99.306 | pass | 3.82 | 2.25 |
|  | 4 | 99.307 | pass | 4.21 | 2.9 |
|  |  |  |  |  |  |
| 105 | 1 | 99.219 | pass | 1.59 | 2.22 |
|  | 2 | 99.221 | fail | 5.62 | 4.11 |
|  | 3 | 99.225 | pass | 2.2 | 2.63 |
|  | 4 | ／ | ／ | ／ | ／ |
|  |  |  |  |  |  |
| 106 | 1 | 97.946 | pass | 2.07 | 1.81 |
|  | 2 | 97.975 | pass | 5.08 | 3.58 |
|  | 3 | 97.998 | pass | 3.76 | 2.58 |
|  | 4 | 98.001 | fail | 5.19 | 3.15 |
|  |  |  |  |  |  |
| 107 | 1 | 97.922 | pass | 3.88 | 2.29 |
|  | 2 | 97.923 | fail | 4.6 | 2.16 |
|  | 3 | 97.924 | pass | 4.82 | 2.73 |
|  | 4 | 97.926 | pass | 4.06 | 2.72 |
|  |  |  |  |  |  |
| 108 | 1 | 99.238 | pass | 2.31 | 2.93 |
|  | 2 | 99.246 | fail | 3.11 | 2.37 |
|  | 3 | 99.249 | fail | 4.23 | 3.17 |
|  | 4 | 99.252 | pass | 4.65 | 2.97 |
|  |  |  |  |  |  |
| 109 | 1 | 99.314 | fail | 4.74 | 2.17 |
|  | 2 | 99.377 | fail | 5.36 | 3.29 |
|  | 3 | ／ | ／ | ／ | ／ |
|  | 4 | ／ | ／ | ／ | ／ |
|  |  |  |  |  |  |
| 110 | 1 | 108.12 | fail | 2.19 | 2.23 |
|  | 2 | 108.13 | fail | 1.96 | 1.85 |
|  | 3 | 108.13 | fail | 3.09 | 2.21 |
|  | 4 | 108.14 | fail | 3.11 | 2.1 |
|  |  |  |  |  |  |
| 111 | 1 | 108.02 | pass | 2.51 | 2.07 |
|  | 2 | 108.03 | pass | 2.71 | 3.24 |
|  | 3 | 108.04 | pass | 3.11 | 2.03 |
|  | 4 | 108.06 | pass | 3.66 | 1.62 |
|  |  |  |  |  |  |
| 112 | 1 | 107.99 | pass | 4.8 | 3.22 |
|  | 2 | 108 | pass | 4.61 | 4.15 |
|  | 3 | 108.01 | fail | 2.42 | 1.96 |
|  | 4 | 108.02 | fail | 2.7 | 2.85 |
|  |  |  |  |  |  |
| 113 | 1 | 108.05 | pass | 2.47 | 1.78 |
|  | 2 | 108.06 | fail | 2.84 | 2.44 |
|  | 3 | 108.06 | fail | 4.58 | 4.08 |
|  | 4 | 108.07 | fail | 1.91 | 1.99 |
|  |  |  |  |  |  |
| 114 | 1 | 108.01 | pass | 4.59 | 2.96 |
|  | 2 | 108.01 | pass | 3.98 | 2.49 |
|  | 3 | 108.02 | fail | 2.79 | 2.6 |
|  | 4 | 108.03 | fail | 3.31 | 2.14 |
|  |  |  |  |  |  |
| 115 | 1 | 108.06 | pass | 2.9 | 1.67 |
|  | 2 | 108.11 | fail | 3.72 | 2.37 |
|  | 3 | 108.38 | fail | 5.1 | 3.01 |
|  | 4 | ／ | ／ | ／ | ／ |
|  |  |  |  |  |  |
| 116 | 1 | 119.12 | pass | 1.94 | 1.81 |
|  | 2 | 119.13 | pass | 2.63 | 1.68 |
|  | 3 | 119.14 | fail | 3.79 | 2.44 |
|  | 4 | 119.14 | fail | 3.71 | 1.89 |
|  |  |  |  |  |  |
| 117 | 1 | 97.927 | pass | 2.72 | 2.37 |
|  | 2 | 97.928 | pass | 3.37 | 2.46 |
|  | 3 | 97.928 | pass | 4.61 | 2.4 |
|  | 4 | 97.929 | pass | 3.23 | 2.19 |
|  |  |  |  |  |  |
| 118 | 1 | 110.65 | pass | 4.65 | 2.98 |
|  | 2 | 110.65 | fail | 3.16 | 2.29 |
|  | 3 | 110.66 | fail | 2.05 | 1.53 |
|  | 4 | 110.66 | fail | 3.4 | 2.23 |
|  |  |  |  |  |  |
| 119 | 1 | 110.61 | pass | 3.27 | 2.52 |
|  | 2 | 110.68 | fail | 4.18 | 3.05 |
|  | 3 | 110.73 | fail | 4.54 | 2.67 |
|  | 4 | ／ | ／ | ／ | ／ |
|  |  |  |  |  |  |
| 120 | 1 | 110.23 | pass | 3.45 | 2.14 |
|  | 2 | 110.25 | fail | 3.63 | 1.81 |
|  | 3 | 110.25 | fail | 3.69 | 3.14 |
|  | 4 | 110.26 | fail | 5.35 | 3.43 |
|  |  |  |  |  |  |
| 121 | 1 | 109.9 | pass | 2.15 | 2.74 |
|  | 2 | 109.91 | fail | 1.7 | 1.67 |
|  | 3 | 109.91 | fail | 2.69 | 1.72 |
|  | 4 | 109.93 | pass | 5.06 | 2.7 |
|  |  |  |  |  |  |
| 122 | 1 | 105.95 | pass | 3.51 | 2.23 |
|  | 2 | 105.95 | fail | 3.21 | 2.4 |
|  | 3 | 106.06 | fail | 4.01 | 3.47 |
|  | 4 | 106.09 | fail | 3.43 | 1.97 |
|  |  |  |  |  |  |
| 123 | 1 | 106.86 | pass | 2.19 | 1.56 |
|  | 2 | 106.86 | pass | 4.36 | 2.61 |
|  | 3 | 106.87 | fail | 3.29 | 2.14 |
|  | 4 | 106.87 | fail | 3.44 | 2.8 |
|  |  |  |  |  |  |
| 124 | 1 | 106.18 | pass | 4.66 | 2.11 |
|  | 2 | 106.22 | fail | 4.41 | 2.66 |
|  | 3 | 106.24 | fail | 4.52 | 4.38 |
|  | 4 | ／ | ／ | ／ | ／ |
|  |  |  |  |  |  |
| 125 | 1 | 111.41 | pass | 2.46 | 1.98 |
|  | 2 | 111.42 | pass | 3.19 | 2.01 |
|  | 3 | 111.42 | pass | 4.03 | 2.91 |
|  | 4 | 111.43 | pass | 2.44 | 1.95 |
|  |  |  |  |  |  |
| 126 | 1 | 107.01 | fail | 4.18 | 2.69 |
|  | 2 | 107.02 | fail | 2.87 | 2.38 |
|  | 3 | 107.02 | pass | 4.14 | 3.32 |
|  | 4 | 107.02 | fail | 2.62 | 2.27 |
|  |  |  |  |  |  |
| 127 | 1 | 110.7 | pass | 1.83 | 2.01 |
|  | 2 | 110.71 | pass | 3.63 | 2.29 |
|  | 3 | 110.71 | fail | 3.89 | 1.85 |
|  | 4 | 110.73 | pass | 4.05 | 2.7 |
|  |  |  |  |  |  |
| 128 | 1 | 110.54 | pass | 3.2 | 2.95 |
|  | 2 | 110.56 | fail | 5.36 | 3.97 |
|  | 3 | 110.66 | pass | 4.09 | 2.55 |
|  | 4 | 110.68 | pass | 4.34 | 2.57 |
|  |  |  |  |  |  |
| 129 | 1 | 110.65 | pass | 5.06 | 3.33 |
|  | 2 | 110.67 | pass | 5.36 | 3.97 |
|  | 3 | 110.67 | pass | 5.57 | 3.56 |
|  | 4 | 110.68 | pass | 5.12 | 3.66 |
|  |  |  |  |  |  |
| 130 | 1 | 110.27 | pass | 5.06 | 3.33 |
|  | 2 | 110.29 | pass | 5.36 | 3.96 |
|  | 3 | 110.35 | pass | 5.57 | 3.56 |
|  | 4 | 110.35 | pass | 5.13 | 3.66 |
|  |  |  |  |  |  |
| 131 | 1 | 110.02 | pass | 4.29 | 3.19 |
|  | 2 | 110.02 | pass | 2.94 | 2.34 |
|  | 3 | 110.03 | pass | 4.08 | 2.47 |
|  | 4 | 110.03 | pass | 4.25 | 3.19 |
|  |  |  |  |  |  |
| 132 | 1 | 110.52 | pass | 2.7 | 2.62 |
|  | 2 | 110.58 | pass | 4.22 | 2.65 |
|  | 3 | 110.58 | fail | 4.56 | 2.46 |
|  | 4 | ／ | ／ | ／ | ／ |
|  |  |  |  |  |  |
| 133 | 1 | 111.91 | pass | 3.6 | 2.36 |
|  | 2 | 111.91 | fail | 3.15 | 2.1 |
|  | 3 | 111.94 | pass | 4.73 | 2.45 |
|  | 4 | 111.95 | fail | 5.43 | 3.13 |
|  |  |  |  |  |  |
| 134 | 1 | 110.77 | pass | 2.98 | 2.26 |
|  | 2 | 110.78 | pass | 4.63 | 3.03 |
|  | 3 | ／ | ／ | ／ | ／ |
|  | 4 | ／ | ／ | ／ | ／ |
|  |  |  |  |  |  |
| 135 | 1 | 110.77 | pass | 4.31 | 2.25 |
|  | 2 | 110.77 | other conforamtion | | |
|  | 3 | 110.89 | fail | 5.44 | 3.22 |
|  | 4 | 110.9 | pass | 4.46 | 2.6 |
|  |  |  |  |  |  |
| 136 | 1 | 110.07 | pass | 3 | 2.74 |
|  | 2 | 110.1 | pass | 4.76 | 2.79 |
|  | 3 | 110.14 | other conforamtion | | |
|  | 4 | 110.14 | pass | 4.07 | 3.05 |
|  |  |  |  |  |  |
| 137 | 1 | 98.15 | pass | 4.37 | 2.47 |
|  | 2 | ／ | ／ | ／ | ／ |
|  | 3 | ／ | ／ | ／ | ／ |
|  | 4 | ／ | ／ | ／ | ／ |
|  |  |  |  |  |  |
| 138 | 1 | 97.94 | pass | 4.51 | 2.78 |
|  | 2 | 97.969 | other conforamtion | | |
|  | 3 | ／ | ／ | ／ | ／ |
|  | 4 | ／ | ／ | ／ | ／ |
|  |  |  |  |  |  |
| 139 | 1 | 97.865 | pass | 2.39 | 1.59 |
|  | 2 | 98.897 | pass | 5 | 3.97 |
|  | 3 | 97.949 | pass | 3.88 | 3.69 |
|  | 4 | 97.972 | other conforamtion | | |
|  |  |  |  |  |  |
| 140 | 1 | 97.776 | pass | 2.02 | 2.25 |
|  | 2 | 97.787 | pass | 4.55 | 2.34 |
|  | 3 | 97.791 | pass | 3.01 | 2.57 |
|  | 4 | 97.805 | pass | 2.49 | 3.03 |
|  |  |  |  |  |  |
| 141 | 1 | 97.497 | fail | 1.98 | 1.65 |
|  | 2 | 97.567 | fail | 5.13 | 2.86 |
|  | 3 | 97.773 | other conforamtion | | |
|  | 4 | 97.81 | other conforamtion | | |
|  |  |  |  |  |  |
| 142 | 1 | 97.446 | fail | 5.18 | 2.42 |
|  | 2 | 97.481 | fail | 3.56 | 2.74 |
|  | 3 | 97.54 | fail | 1.33 | 1.46 |
|  | 4 | 97.54 | other conforamtion | | |
|  |  |  |  |  |  |
| 143 | 1 | 97.962 | pass | 4.45 | 3.77 |
|  | 2 | 97.964 | pass | 4.59 | 2.69 |
|  | 3 | 97.968 | pass | 3.27 | 2.74 |
|  | 4 | 97.978 | pass | 3.83 | 3.34 |
|  |  |  |  |  |  |
| 144 | 1 | 97.943 | fail | 4.42 | 2.14 |
|  | 2 | 97.945 | fail | 3.32 | 2.85 |
|  | 3 | 97.948 | fail | 3.14 | 2.56 |
|  | 4 | 97.952 | fail | 4.13 | 2.9 |
|  |  |  |  |  |  |
| 145 | 1 | 97.936 | pass | 3.11 | 2.42 |
|  | 2 | 97.937 | pass | 4.19 | 2.91 |
|  | 3 | 97.943 | pass | 2.56 | 2.75 |
|  | 4 | 97.953 | pass | 4.39 | 2.87 |
|  |  |  |  |  |  |
| 146 | 1 | 97.993 | pass | 2.84 | 1.7 |
|  | 2 | 97.993 | pass | 3.77 | 2.74 |
|  | 3 | 98.024 | pass | 3.66 | 1.91 |
|  | 4 | 98.065 | pass | 3.58 | 2.01 |
|  |  |  |  |  |  |
| 147 | 1 | 108.32 | pass | 4.32 | 3.15 |
|  | 2 | 108.33 | pass | 3.84 | 2.36 |
|  | 3 | 108.33 | pass | 2.85 | 1.84 |
|  | 4 | 108.33 | pass | 3.58 | 2.62 |
|  |  |  |  |  |  |
| 148 | 1 | 108.42 | pass | 4.76 | 3.88 |
|  | 2 | 108.42 | pass | 2.89 | 2.43 |
|  | 3 | 108.43 | pass | 4.01 | 2.65 |
|  | 4 | 108.45 | pass | 4 | 2.58 |
|  |  |  |  |  |  |
| 149 | 1 | 108.42 | pass | 3.03 | 2.34 |
|  | 2 | 108.43 | pass | 4.02 | 2.21 |
|  | 3 | 108.43 | pass | 4.47 | 2.7 |
|  | 4 | 108.45 | pass | 4.53 | 2.39 |
|  |  |  |  |  |  |
| 150 | 1 | 108.35 | pass | 2.67 | 1.43 |
|  | 2 | 108.36 | pass | 4.46 | 3.34 |
|  | 3 | 108.37 | fail | 4.66 | 3.65 |
|  | 4 | 108.39 | fail | 3.77 | 2.45 |
|  |  |  |  |  |  |
| 151 | 1 | 98.622 | fail | 5.36 | 2.4 |
|  | 2 | 98.622 | fail | 4.14 | 2.7 |
|  | 3 | 98.623 | other conforamtion | | |
|  | 4 | 98.645 | fail | 4.69 | 2.32 |
|  |  |  |  |  |  |
| 152 | 1 | 98.61 | pass | 2.94 | 1.91 |
|  | 2 | 98.615 | pass | 3.24 | 1.96 |
|  | 3 | 98.642 | pass | 5.26 | 2.46 |
|  | 4 | 98.645 | pass | 3.11 | 2.01 |
|  |  |  |  |  |  |
| 153 | 1 | 98.519 | fail | 4.67 | 2.88 |
|  | 2 | 98.521 | fail | 5.7 | 2.99 |
|  | 3 | 98.531 | fail | 5.68 | 3.84 |
|  | 4 | 98.542 | fail | 5.23 | 1.85 |
|  |  |  |  |  |  |
| 154 | 1 | 98.495 | pass | 4.59 | 3.89 |
|  | 2 | 98.498 | fail | 2.67 | 3.12 |
|  | 3 | 98.498 | fail | 3.74 | 3.17 |
|  | 4 | 98.501 | fail | 4.12 | 2.13 |
|  |  |  |  |  |  |
| 155 | 1 | 98.5 | pass | 2.95 | 2.73 |
|  | 2 | 98.5 | pass | 4.33 | 2.88 |
|  | 3 | 98.501 | pass | 4.83 | 2.33 |
|  | 4 | 98.503 | fail | 5.26 | 3.43 |
|  |  |  |  |  |  |
| 156 | 1 | 98.498 | pass | 2.34 | 2.03 |
|  | 2 | 98.5 | pass | 3.26 | 2.58 |
|  | 3 | 98.5 | fail | 2.63 | 2.12 |
|  | 4 | 98.504 | other conforamtion | | |
|  |  |  |  |  |  |
| 157 | 1 | 98.501 | pass | 3.43 | 1.69 |
|  | 2 | 98.502 | pass | 2.85 | 2.46 |
|  | 3 | 98.504 | pass | 3.64 | 2.45 |
|  | 4 | 98.519 | pass | 4.46 | 2.93 |
|  |  |  |  |  |  |
| 158 | 1 | 98.462 | pass | 5.11 | 2.59 |
|  | 2 | 98.479 | fail | 2.74 | 1.99 |
|  | 3 | 98.511 | fail | 3.68 | 1.87 |
|  | 4 | 98.522 | fail | 2.83 | 1.96 |
|  |  |  |  |  |  |
| 159 | 1 | 98.463 | pass | 1.43 | 1.43 |
|  | 2 | 98.483 | pass | 5.62 | 3.95 |
|  | 3 | 98.537 | pass | 2.15 | 2.03 |
|  | 4 | 98.572 | fail | 4.65 | 3.01 |
|  |  |  |  |  |  |
| 160 | 1 | 97.801 | pass | 2.44 | 2.05 |
|  | 2 | 97.809 | pass | 2.66 | 2.15 |
|  | 3 | 97.817 | pass | 4.63 | 2.15 |
|  | 4 | 97.817 | pass | 4.01 | 2.47 |
|  |  |  |  |  |  |
| 161 | 1 | 98.243 | pass | 2.69 | 1.93 |
|  | 2 | 98.25 | pass | 2.97 | 2.02 |
|  | 3 | 98.252 | pass | 3.6 | 2.18 |
|  | 4 | 98.252 | pass | 4.56 | 2.62 |
|  |  |  |  |  |  |
| 162 | 1 | 99.417 | pass | 2.75 | 3.28 |
|  | 2 | 99.483 | pass | 6.12 | 3.38 |
|  | 3 | 99.492 | fail | 5.31 | 2.8 |
|  | 4 | 99.498 | pass | 3.86 | 1.69 |
|  |  |  |  |  |  |
| 163 | 1 | 99.453 | pass | 1.76 | 1.39 |
|  | 2 | 99.478 | pass | 4.88 | 2.82 |
|  | 3 | 99.497 | pass | 4.49 | 3.26 |
|  | 4 | 99.52 | pass | 5.63 | 3.31 |
|  |  |  |  |  |  |
| 164 | 1 | 98.273 | pass | 4.44 | 2.89 |
|  | 2 | 98.276 | pass | 4.49 | 2.86 |
|  | 3 | 98.328 | pass | 5.34 | 3.21 |
|  | 4 | 98.302 | pass | 5.98 | 3.63 |
|  |  |  |  |  |  |
| 165 | 1 | 97.791 | pass | 2.98 | 2.73 |
|  | 2 | 97.792 | pass | 3.91 | 3.01 |
|  | 3 | 97.795 | pass | 4.12 | 2.07 |
|  | 4 | 97.797 | pass | 3.19 | 3.01 |
|  |  |  |  |  |  |
| 166 | 1 | 97.883 | pass | 3.46 | 2.82 |
|  | 2 | 97.889 | pass | 4.04 | 2.79 |
|  | 3 | 97.913 | pass | 4.95 | 2.36 |
|  | 4 | 97.915 | pass | 4.25 | 2.13 |
|  |  |  |  |  |  |
| 167 | 1 | 97.79 | pass | 3.65 | 1.71 |
|  | 2 | 97.79 | pass | 4.7 | 2.74 |
|  | 3 | 97.792 | pass | 3.75 | 2.52 |
|  | 4 | 97.792 | pass | 4.67 | 2.19 |
|  |  |  |  |  |  |
| 168 | 1 | 97.787 | pass | 3.3 | 2.45 |
|  | 2 | 97.789 | pass | 4.04 | 2.46 |
|  | 3 | 97.792 | pass | 5.49 | 2.95 |
|  | 4 | 79.797 | pass | 4.29 | 2.6 |
|  |  |  |  |  |  |
| 169 | 1 | 97.721 | pass | 2.16 | 1.78 |
|  | 2 | 97.746 | pass | 5.48 | 3 |
|  | 3 | 97.768 | pass | 3.74 | 2.96 |
|  | 4 | 97.791 | pass | 4.34 | 2.31 |
|  |  |  |  |  |  |
| 170 | 1 | 97.785 | pass | 3.5 | 1.6 |
|  | 2 | 97.788 | pass | 5.15 | 2.65 |
|  | 3 | 97.791 | pass | 3.55 | 1.86 |
|  | 4 | 97.794 | pass | 4.47 | 2.76 |
|  |  |  |  |  |  |
| 171 | 1 | 98.109 | pass | 4.12 | 2.11 |
|  | 2 | 98.11 | fail | 4.83 | 2.35 |
|  | 3 | ／ | ／ | ／ | ／ |
|  | 4 | ／ | ／ | ／ | ／ |
|  |  |  |  |  |  |
| 172 | 1 | 97.789 | pass | 2.58 | 2.09 |
|  | 2 | 97.789 | pass | 5.41 | 2.8 |
|  | 3 | 97.793 | pass | 3.36 | 1.97 |
|  | 4 | 97.795 | pass | 5.12 | 2.19 |
|  |  |  |  |  |  |
| 173 | 1 | 97.912 | pass | 2.9 | 2.35 |
|  | 2 | 97.914 | pass | 3.76 | 2.82 |
|  | 3 | 97.915 | pass | 4.92 | 2.76 |
|  | 4 | 97.915 | pass | 5.35 | 2.57 |
|  |  |  |  |  |  |
| 174 | 1 | 97.783 | pass | 3.43 | 2.42 |
|  | 2 | 97.788 | pass | 4.22 | 2.07 |
|  | 3 | 97.792 | pass | 4.49 | 2.43 |
|  | 4 | 97.793 | pass | 4.36 | 2.17 |
|  |  |  |  |  |  |
| 175 | 1 | 97.794 | pass | 2.65 | 2.51 |
|  | 2 | 97.797 | pass | 5.33 | 2.78 |
|  | 3 | 97.797 | pass | 4.89 | 2.46 |
|  | 4 | 97.806 | pass | 3.23 | 2.24 |
|  |  |  |  |  |  |
| 176 | 1 | 97.777 | pass | 2.38 | 1.94 |
|  | 2 | 97.789 | pass | 4.61 | 2.01 |
|  | 3 | 97.791 | pass | 2.97 | 1.89 |
|  | 4 | 97.797 | pass | 4.71 | 2.7 |
|  |  |  |  |  |  |
| 177 | 1 | 98.291 | pass | 3.67 | 2.64 |
|  | 2 | 98.291 | pass | 4.17 | 2.62 |
|  | 3 | 98.292 | pass | 5.44 | 5.63 |
|  | 4 | 98.297 | pass | 4.44 | 2.98 |
|  |  |  |  |  |  |
| 178 | 1 | 97.796 | pass | 2.58 | 2.38 |
|  | 2 | 97.797 | pass | 3.11 | 2.51 |
|  | 3 | 79.798 | pass | 5.3 | 2.56 |
|  | 4 | 97.798 | pass | 3.82 | 2.95 |
|  |  |  |  |  |  |
| 179 | 1 | 97.932 | pass | 2.56 | 2.17 |
|  | 2 | 97.934 | pass | 3.14 | 2.13 |
|  | 3 | 97.941 | fail | 3.17 | 2.45 |
|  | 4 | 97.946 | pass | 3.31 | 2.23 |
|  |  |  |  |  |  |
| 180 | 1 | 106.29 | pass | 1.81 | 1.97 |
|  | 2 | 106.36 | pass | 4.71 | 2.28 |
|  | 3 | 106.36 | pass | 4.72 | 3.22 |
|  | 4 | 106.36 | pass | 3.35 | 2.45 |
|  |  |  |  |  |  |
| 181 | 1 | 115.89 | pass | 4.38 | 2.24 |
|  | 2 | ／ | ／ | ／ | ／ |
|  | 3 | ／ | ／ | ／ | ／ |
|  | 4 | ／ | ／ | ／ | ／ |
|  |  |  |  |  |  |
| 182 | 1 | 116.06 | pass | 4.06 | 2.6 |
|  | 2 | 116.07 | pass | 4.83 | 2.8 |
|  | 3 | 116.09 | pass | 4.6 | 2.21 |
|  | 4 | 116.09 | pass | 4.95 | 2.74 |
|  |  |  |  |  |  |
| 183 | 1 | 115.96 | pass | 2.56 | 2.19 |
|  | 2 | 115.96 | pass | 4.76 | 2.6 |
|  | 3 | 115.97 | pass | 5.01 | 2.4 |
|  | 4 | 116 | pass | 4.21 | 2.24 |
|  |  |  |  |  |  |
| 184 | 1 | 115.9 | pass | 2.22 | 2.01 |
|  | 2 | 115.93 | pass | 4.15 | 2.54 |
|  | 3 | 115.94 | pass | 3.72 | 1.95 |
|  | 4 | 115.96 | other conforamtion | | |
|  |  |  |  |  |  |
| 185 | 1 | 115.5 | fail | 4.31 | 2.51 |
|  | 2 | ／ | ／ | ／ | ／ |
|  | 3 | ／ | ／ | ／ | ／ |
|  | 4 | ／ | ／ | ／ | ／ |
|  |  |  |  |  |  |
| 186 | 1 | 116.21 | pass | 4.96 | 2.68 |
|  | 2 | 116.22 | pass | 5.17 | 3.48 |
|  | 3 | 116.28 | fail | 4.14 | 3.26 |
|  | 4 | 116.33 | fail | 3.76 | 2.11 |
|  |  |  |  |  |  |
| 187 | 1 | 115.96 | pass | 4.56 | 2.78 |
|  | 2 | 115.99 | pass | 4.85 | 3.5 |
|  | 3 | 116 | pass | 5.24 | 2.92 |
|  | 4 | 116.02 | pass | 5.17 | 2.59 |
|  |  |  |  |  |  |
| 188 | 1 | 97.879 | pass | 3.48 | 2.47 |
|  | 2 | 97.88 | fail | 3.87 | 2.71 |
|  | 3 | 97.884 | fail | 3.26 | 2.38 |
|  | 4 | 97.886 | fail | 3.95 | 2 |
|  |  |  |  |  |  |
| 189 | 1 | 97.891 | pass | 2.62 | 2.25 |
|  | 2 | 97.894 | pass | 2.68 | 2.58 |
|  | 3 | 97.929 | fail | 2.16 | 1.63 |
|  | 4 | 97.935 | fail | 4.5 | 2.78 |
|  |  |  |  |  |  |
| 190 | 1 | 98.08 | pass | 2.58 | 1.73 |
|  | 2 | 98.1 | fail | 3.57 | 1.64 |
|  | 3 | 98.103 | other conforamtion | | |
|  | 4 | 98.108 | pass | 3.26 | 2.6 |
|  |  |  |  |  |  |
| 191 | 1 | 97.868 | pass | 2.78 | 2.73 |
|  | 2 | 97.872 | pass | 2.93 | 1.63 |
|  | 3 | 97.891 | pass | 3.12 | 2.68 |
|  | 4 | 97.892 | fail | 4.72 | 2.13 |
|  |  |  |  |  |  |
| 192 | 1 | 98.402 | pass | 2.93 | 1.63 |
|  | 2 | 98.405 | fail | 2.78 | 2.73 |
|  | 3 | 98.419 | pass | 3.12 | 2.68 |
|  | 4 | 98.424 | other conforamtion | | |
|  |  |  |  |  |  |
| 193 | 1 | 98.35 | pass | 3.92 | 2.4 |
|  | 2 | 98.352 | pass | 4.8 | 2.98 |
|  | 3 | 98.353 | pass | 4.28 | 1.76 |
|  | 4 | 98.369 | pass | 4.44 | 2.81 |
|  |  |  |  |  |  |
| 194 | 1 | 98.344 | pass | 4.05 | 2.84 |
|  | 2 | 98.351 | pass | 5.41 | 2.05 |
|  | 3 | 98.393 | pass | 4.94 | 2.48 |
|  | 4 | ／ | ／ | ／ | ／ |
|  |  |  |  |  |  |
| 195 | 1 | 98.531 | pass | 3.63 | 2.08 |
|  | 2 | 98.549 | pass | 4.49 | 1.35 |
|  | 3 | 98.577 | pass | 4.33 | 2.69 |
|  | 4 | 98.589 | fail | 3.96 | 1.84 |
|  |  |  |  |  |  |
| 196 | 1 | 98.343 | pass | 2.81 | 2.3 |
|  | 2 | 98.41 | pass | 3.64 | 1.51 |
|  | 3 | 98.475 | other conforamtion | | |
|  | 4 | 98.545 | pass | 5.03 | 2.85 |
|  |  |  |  |  |  |
| 197 | 1 | 98.962 | pass | 4.17 | 2.55 |
|  | 2 | 98.963 | pass | 4.49 | 2.77 |
|  | 3 | 98.969 | fail | 3.5 | 1.49 |
|  | 4 | 98.983 | pass | 5.8 | 2.98 |
|  |  |  |  |  |  |
| 198 | 1 | 97.381 | fail | 1.55 | 2.15 |
|  | 2 | 97.383 | fail | 4.24 | 2.86 |
|  | 3 | 97.385 | fail | 3.19 | 1.77 |
|  | 4 | 97.435 | fail | 2.92 | 2.28 |
|  |  |  |  |  |  |
| 199 | 1 | 97.831 | pass | 3.45 | 2.01 |
|  | 2 | 97.82 | pass | 4.23 | 2.62 |
|  | 3 | 97.851 | pass | 4.32 | 2.13 |
|  | 4 | 97.855 | pass | 3.91 | 1.7 |
|  |  |  |  |  |  |
| 200 | 1 | 97.837 | pass | 3.51 | 1.68 |
|  | 2 | 97.844 | pass | 5.06 | 2.25 |
|  | 3 | 97.846 | pass | 5.81 | 2.87 |
|  | 4 | 97.853 | pass | 3.81 | 1.74 |
|  |  |  |  |  |  |
| 201 | 1 | 97.805 | pass | 3.65 | 2.95 |
|  | 2 | 97.828 | pass | 4.23 | 2.99 |
|  | 3 | 97.871 | pass | 4.63 | 2.62 |
|  | 4 | ／ | ／ | ／ | ／ |
|  |  |  |  |  |  |
| 202 | 1 | 97.786 | pass | 3.22 | 2.89 |
|  | 2 | 97.787 | pass | 4.16 | 3.26 |
|  | 3 | 97.789 | pass | 4.05 | 2.78 |
|  | 4 | 97.798 | pass | 4.6 | 3.44 |
|  |  |  |  |  |  |
| 203 | 1 | 97.821 | pass | 3.53 | 2.09 |
|  | 2 | 97.839 | pass | 4.94 | 2.41 |
|  | 3 | 97.842 | pass | 4.59 | 2.34 |
|  | 4 | 97.842 | pass | 4.84 | 2.3 |
|  |  |  |  |  |  |
| 204 | 1 | 97.776 | pass | 2.33 | 1.72 |
|  | 2 | 97.777 | pass | 2.6 | 2.87 |
|  | 3 | 97.779 | pass | 4.17 | 3.18 |
|  | 4 | 97.783 | pass | 3.18 | 2.93 |
|  |  |  |  |  |  |
| 205 | 1 | 97.772 | pass | 2.99 | 2.24 |
|  | 2 | 97.779 | pass | 4.64 | 3.5 |
|  | 3 | 97.816 | pass | 5.52 | 3.41 |
|  | 4 | 97.827 | fail | 5.46 | 3.52 |
|  |  |  |  |  |  |
| 206 | 1 | 97.833 | pass | 3.51 | 2.57 |
|  | 2 | 97.838 | pass | 4.77 | 2.11 |
|  | 3 | 97.841 | fail | 3.9 | 2.59 |
|  | 4 | 97.842 | pass | 5.28 | 2.37 |
|  |  |  |  |  |  |
| 207 | 1 | 97.877 | fail | 5.22 | 2.8 |
|  | 2 | ／ | ／ | ／ | ／ |
|  | 3 | ／ | ／ | ／ | ／ |
|  | 4 | ／ | ／ | ／ | ／ |
|  |  |  |  |  |  |
| 208 | 1 | 101.38 | pass | 1.62 | 1.64 |
|  | 2 | 101.42 | fail | 2.63 | 2.11 |
|  | 3 | 101.43 | pass | 4.33 | 2.73 |
|  | 4 | 101.48 | pass | 4.31 | 2.87 |
|  |  |  |  |  |  |
| 209 | 1 | 97.835 | pass | 4.5 | 3.93 |
|  | 2 | 97.853 | fail | 3.49 | 2.4 |
|  | 3 | 97.885 | pass | 3.41 | 2.24 |
|  | 4 | 97.843 | pass | 2.33 | 1.97 |
|  |  |  |  |  |  |
| 210 | 1 | 97.731 | pass | 4.63 | 3.45 |
|  | 2 | 97.747 | fail | 3.93 | 2.46 |
|  | 3 | 97.779 | pass | 2.63 | 2.69 |
|  | 4 | 97.781 | fail | 3.11 | 1.88 |
|  |  |  |  |  |  |
| 211 | 1 | 97.725 | pass | 4.91 | 3.49 |
|  | 2 | 97.739 | pass | 4.24 | 3.32 |
|  | 3 | 97.739 | pass | 2.45 | 2.53 |
|  | 4 | 97.944 | fail | 4.92 | 2.82 |
|  |  |  |  |  |  |
| 212 | 1 | 97.881 | pass | 6.18 | 3.47 |
|  | 2 | 97.883 | pass | 1.87 | 2.01 |
|  | 3 | 97.885 | fail | 2.89 | 1.55 |
|  | 4 | 97.924 | fail | 3.58 | 1.93 |
|  |  |  |  |  |  |
| 213 | 1 | 97.974 | pass | 4.88 | 3.42 |
|  | 2 | 98.063 | pass | 3.48 | 2.41 |
|  | 3 | ／ | ／ | ／ | ／ |
|  | 4 | ／ | ／ | ／ | ／ |
|  |  |  |  |  |  |
| 214 | 1 | 98.025 | pass | 3.6 | 2.6 |
|  | 2 | ／ | ／ | ／ | ／ |
|  | 3 | ／ | ／ | ／ | ／ |
|  | 4 | ／ | ／ | ／ | ／ |
|  |  |  |  |  |  |
| 215 | 1 | 97.99 | pass | 3.87 | 2.93 |
|  | 2 | 97.998 | pass | 3.99 | 2.96 |
|  | 3 | 98.033 | pass | 4.21 | 3.6 |
|  | 4 | 98.045 | pass | 4.44 | 2.83 |
|  |  |  |  |  |  |
| 216 | 1 | 98.065 | pass | 3.08 | 2.73 |
|  | 2 | ／ | ／ | ／ | ／ |
|  | 3 | ／ | ／ | ／ | ／ |
|  | 4 | ／ | ／ | ／ | ／ |
|  |  |  |  |  |  |
| 217 | 1 | 97.774 | pass | 3.21 | 2.21 |
|  | 2 | 97.777 | pass | 3.52 | 2.02 |
|  | 3 | 97.819 | fail | 4.22 | 2.21 |
|  | 4 | ／ | ／ | ／ | ／ |
|  |  |  |  |  |  |
| 218 | 1 | 97.75 | pass | 3.5 | 2.36 |
|  | 2 | 97.753 | pass | 3.6 | 3.53 |
|  | 3 | 97.764 | pass | 4.5 | 2.4 |
|  | 4 | 97.766 | fail | 5.43 | 3.45 |
|  |  |  |  |  |  |
| 219 | 1 | 97.822 | pass | 3.25 | 2.06 |
|  | 2 | 97.836 | pass | 4.78 | 2.75 |
|  | 3 | 97.877 | fail | 4.35 | 2.91 |
|  | 4 | ／ | ／ | ／ | ／ |
|  |  |  |  |  |  |
| 220 | 1 | 97.902 | pass | 3.92 | 2.02 |
|  | 2 | 97.816 | fail | 4.39 | 2.64 |
|  | 3 | 97.871 | fail | 6.04 | 3.11 |
|  | 4 | ／ | ／ | ／ | ／ |
|  |  |  |  |  |  |
| 221 | 1 | 97.884 | pass | 2.75 | 1.84 |
|  | 2 | ／ | ／ | ／ | ／ |
|  | 3 | ／ | ／ | ／ | ／ |
|  | 4 | ／ | ／ | ／ | ／ |
|  |  |  |  |  |  |
| 222 | 1 | 97.97 | pass | 2.83 | 1.4 |
|  | 2 | 98.1 | pass | 3.67 | 2.02 |
|  | 3 | 98.988 | pass | 5.59 | 2.68 |
|  | 4 | 99.012 | other conforamtion | | |
|  |  |  |  |  |  |
| 223 | 1 | 97.841 | fail | 4.28 | 2.64 |
|  | 2 | ／ | ／ | ／ | ／ |
|  | 3 | ／ | ／ | ／ | ／ |
|  | 4 | ／ | ／ | ／ | ／ |
|  |  |  |  |  |  |
| 224 | 1 | 97.524 | pass | 5.51 | 2.55 |
|  | 2 | 97.535 | fail | 4.23 | 2.94 |
|  | 3 | 97.537 | fail | 2.15 | 2.41 |
|  | 4 | 97.554 | fail | 2.85 | 2.61 |
|  |  |  |  |  |  |
| 225 | 1 | 97.813 | pass | 3.98 | 2.08 |
|  | 2 | 97.825 | pass | 2.9 | 2.23 |
|  | 3 | ／ | ／ | ／ | ／ |
|  | 4 | ／ | ／ | ／ | ／ |
|  |  |  |  |  |  |
| 226 | 1 | 97.628 | pass | 1.6 | 2.11 |
|  | 2 | 97.644 | other conforamtion | | |
|  | 3 | 97.652 | fail | 4.63 | 2.86 |
|  | 4 | 97.663 | other conforamtion | | |
|  |  |  |  |  |  |
| 227 | 1 | 97.808 | pass | 1.67 | 1.54 |
|  | 2 | 97.823 | other conforamtion | | |
|  | 3 | 97.92 | pass | 4.11 | 2.7 |
|  | 4 | ／ | ／ | ／ | ／ |
|  |  |  |  |  |  |
| 228 | 1 | 97.625 | pass | 1.78 | 1.76 |
|  | 2 | 97.636 | pass | 3.49 | 1.96 |
|  | 3 | 97.694 | pass | 3.13 | 1.99 |
|  | 4 | ／ | ／ | ／ | ／ |
|  |  |  |  |  |  |
| 229 | 1 | 97.869 | pass | 3.63 | 2.94 |
|  | 2 | 98.027 | pass | 3.87 | 2.8 |
|  | 3 | 98.036 | pass | 5.01 | 3.6 |
|  | 4 | 98.188 | pass | 4.16 | 2.23 |
|  |  |  |  |  |  |
| 230 | 1 | 97.739 | pass | 3.6 | 2.95 |
|  | 2 | 97.739 | other conforamtion | | |
|  | 3 | 97.744 | other conforamtion | | |
|  | 4 | 97.755 | other conforamtion | | |
|  |  |  |  |  |  |
| 231 | 1 | 100.32 | pass | 3.4 | 2.43 |
|  | 2 | 100.33 | fail | 3.37 | 3.3 |
|  | 3 | 100.35 | fail | 4.27 | 3.37 |
|  | 4 | 100.38 | fail | 4.99 | 3.21 |
|  |  |  |  |  |  |
| 232 | 1 | 100.11 | pass | 2.98 | 2.88 |
|  | 2 | 100.11 | pass | 3.09 | 2.76 |
|  | 3 | 100.12 | pass | 4.75 | 3.16 |
|  | 4 | 100.24 | pass | 4.57 | 2.92 |
|  |  |  |  |  |  |
| 233 | 1 | 102.48 | pass | 3.58 | 2.31 |
|  | 2 | 102.49 | pass | 4.48 | 2.5 |
|  | 3 | 102.49 | pass | 4.59 | 2.39 |
|  | 4 | 102.49 | pass | 4.91 | 2.75 |

**Screening result of CASS**

Table S2 Screening information of each compound

| Compound | Conformational check result | Distance 1 (Å) | Distance 2 (Å) | Accepted or rejected by CASS | Reaction with enzyme happen or not reported by reference | Reference number of the compounds |
| --- | --- | --- | --- | --- | --- | --- |
| 1 | pass | 3.28 | 2.23 | accepted | yes | 1 |
| 2 | pass | 6.39 | 4.68 | rejected | no | 1 |
| 3 | pass | 2.83 | 2.51 | accepted | yes | 1 |
| 4 | pass | 3.74 | 3.29 | rejected | no | 1 |
| 5 | pass | 2.66 | 2.09 | accepted | yes | 1 |
| 6 | fail | 3.06 | 2.53 | rejected | no | 1 |
| 7 | pass | 2.5 | 2.32 | accepted | yes | 1 |
| 8 | pass | 4.29 | 3.31 | rejected | no | 1 |
| 9 | pass | 3.85 | 2.65 | accepted | yes | 1 |
| 10 | pass | 5.12 | 3.59 | rejected | no | 1 |
| 11 | pass | 2.76 | 3.14 | accepted | yes | 1 |
| 12 | pass | 3.74 | 3.24 | rejected | no | 2 |
| 13 | pass | 2.37 | 1.69 | accepted | yes | 2 |
| 14 | pass | 4.2 | 3.53 | rejected | no | 3 |
| 15 | pass | 2.2 | 1.94 | accepted | yes | 3 |
| 16 | fail | 3.07 | 2.05 | rejected | no | 3 |
| 17 | pass | 3.06 | 2.86 | accepted | yes | 3 |
| 18 | pass | 1.98 | 2.81 | accepted | yes | 4 |
| 19 | pass | 3.04 | 2.8 | accepted | yes | 4 |
| 20 | pass | 3.41 | 1.86 | accepted | yes | 4 |
| 21 | pass | 2.75 | 1.81 | accepted | yes | 4 |
| 22 | pass | 2.72 | 1.22 | accepted | yes | 4 |
| 23 | pass | 4.44 | 2.54 | accepted | yes | 4 |
| 24 | pass | 1.34 | 2.56 | accepted | yes | 4 |
| 25 | fail | 4.32 | 2.28 | rejected | no | 5 |
| 26 | pass | 2.73 | 2.08 | accepted | yes | 5 |
| 27 | fail | 2.02 | 2.31 | rejected | no | 5 |
| 28 | pass | 2.89 | 2.15 | accepted | yes | 5 |
| 29 | pass | 5.54 | 3.08 | accepted | yes | 6 |
| 30 | pass | 3.65 | 3.03 | accepted | yes | 6 |
| 31 | pass | 2.89 | 3.15 | rejected | no | 6 |
| 32 | pass | 3.69 | 2.59 | accepted | yes | 6 |
| 33 | pass | 3.51 | 3.41 | rejected | no | 6 |
| 34 | pass | 2.57 | 1.99 | accepted | yes | 6 |
| 35 | pass | 2.36 | 1.72 | accepted | yes | 6 |
| 36 | pass | 3.5 | 3.13 | rejected | no | 6 |
| 37 | pass | 4.3 | 3.17 | rejected | no | 6 |
| 38 | pass | 3.69 | 2.14 | accepted | yes | 6 |
| 39 | fail | 3.08 | 2.21 | rejected | no | 6 |
| 40 | fail | 3.99 | 2.03 | rejected | no | 6 |
| 41 | fail | 2.66 | 1.72 | rejected | no | 6 |
| 42 | pass | 2.9 | 3.35 | rejected | no | 6 |
| 43 | pass | 2.94 | 2.37 | accepted | yes | 7 |
| 44 | pass | 1.29 | 2.49 | accepted | yes | 7 |
| 45 | pass | 4.93 | 3.05 | accepted | yes | 7 |
| 46 | pass | 3.13 | 2.95 | accepted | yes | 7 |
| 47 | pass | 3.09 | 1.64 | accepted | yes | 8 |
| 48 | pass | 5.2 | 3.01 | accepted | yes | 8 |
| 49 | pass | 3.16 | 2.08 | accepted | yes | 8 |
| 50 | pass | 4.57 | 2.61 | accepted | yes | 8 |
| 51 | pass | 2.75 | 1.62 | accepted | yes | 8 |
| 52 | pass | 3.24 | 2.88 | accepted | yes | 9 |
| 53 | pass | 2.17 | 2.23 | accepted | yes | 10 |
| 54 | pass | 2.47 | 2.02 | accepted | yes | 10 |
| 55 | pass | 2.72 | 2.26 | accepted | yes | 10 |
| 56 | pass | 2.9 | 2.94 | accepted | yes | 10 |
| 57 | pass | 2.28 | 1.83 | accepted | yes | 11 |
| 58 | pass | 4.48 | 2.99 | accepted | yes | 12 |
| 59 | pass | 2.55 | 3.25 | accepted | yes | 12 |
| 60 | pass | 3.97 | 2.78 | accepted | yes | 12 |
| 61 | pass | 3.22 | 2.75 | accepted | yes | 12 |
| 62 | pass | 2.89 | 2.29 | accepted | yes | 13 |
| 63 | pass | 3.69 | 2.31 | accepted | yes | 13 |
| 64 | pass | 2.08 | 1.97 | accepted | yes | 13 |
| 65 | pass | 4.42 | 2.98 | accepted | yes | 13 |
| 66 | pass | 2.3 | 2.17 | accepted | yes | 13 |
| 67 | pass | 2.33 | 1.52 | accepted | yes | 13 |
| 68 | pass | 3.03 | 2.72 | accepted | yes | 13 |
| 69 | pass | 2.43 | 2.97 | accepted | yes | 13 |
| 70 | pass | 2.33 | 2.52 | accepted | yes | 13 |
| 71 | pass | 3.47 | 2.73 | accepted | yes | 13 |
| 72 | pass | 1.96 | 2.15 | accepted | yes | 13 |
| 73 | pass | 2.2 | 1.56 | accepted | yes | 13 |
| 74 | pass | 3.77 | 2.1 | accepted | yes | 14 |
| 75 | pass | 3.63 | 2.06 | accepted | yes | 14 |
| 76 | pass | 3.83 | 2.27 | accepted | yes | 15 |
| 77 | pass | 2.53 | 2.08 | accepted | yes | 15 |
| 78 | pass | 2.69 | 1.74 | accepted | yes | 15 |
| 79 | pass | 4.2 | 2.59 | accepted | yes | 16 |
| 80 | pass | 2.08 | 1.4 | accepted | yes | 16 |
| 81 | pass | 3.21 | 1.64 | accepted | yes | 16 |
| 82 | pass | 2.03 | 2.2 | accepted | yes | 16 |
| 83 | pass | 3.19 | 2.76 | accepted | yes | 17 |
| 84 | pass | 4.15 | 2.34 | accepted | yes | 18 |
| 85 | pass | 4.09 | 1.92 | accepted | yes | 18 |
| 86 | pass | 3.56 | 2.13 | accepted | yes | 18 |
| 87 | pass | 2.59 | 2.05 | accepted | yes | 18 |
| 88 | pass | 3.2 | 1.98 | accepted | yes | 18 |
| 89 | pass | 3.38 | 2.5 | accepted | yes | 18 |
| 90 | pass | 2.22 | 1.8 | accepted | yes | 18 |
| 91 | pass | 2.99 | 2.34 | accepted | yes | 18 |
| 92 | pass | 3.78 | 2.78 | accepted | yes | 18 |
| 93 | pass | 3.78 | 1.88 | accepted | yes | 19 |
| 94 | pass | 2.72 | 2.49 | accepted | yes | 19 |
| 95 | pass | 1.69 | 2.43 | accepted | yes | 19 |
| 96 | pass | 3.04 | 2.16 | accepted | yes | 19 |
| 97 | pass | 3.49 | 1.95 | accepted | yes | 19 |
| 98 | pass | 4.07 | 2.67 | accepted | yes | 19 |
| 99 | pass | 2.64 | 2.02 | accepted | yes | 19 |
| 100 | pass | 2.37 | 2.25 | accepted | yes | 19 |
| 101 | pass | 2.06 | 2.15 | accepted | yes | 19 |
| 102 | pass | 3.01 | 2.26 | accepted | yes | 19 |
| 103 | pass | 2.41 | 1.9 | accepted | yes | 19 |
| 104 | pass | 1.88 | 1.79 | accepted | yes | 19 |
| 105 | pass | 1.59 | 2.22 | accepted | yes | 19 |
| 106 | pass | 2.07 | 1.81 | accepted | yes | 19 |
| 107 | pass | 3.88 | 2.29 | accepted | yes | 19 |
| 108 | pass | 2.31 | 2.93 | accepted | yes | 19 |
| 109 | fail | 4.74 | 2.17 | rejected | yes | 19 |
| 110 | fail | 2.19 | 2.23 | rejected | no | 20 |
| 111 | pass | 2.51 | 2.07 | accepted | yes | 20 |
| 112 | pass | 4.8 | 3.22 | rejected | no | 20 |
| 113 | pass | 2.47 | 1.78 | accepted | yes | 20 |
| 114 | pass | 4.59 | 2.96 | accepted | yes | 20 |
| 115 | pass | 2.9 | 1.67 | accepted | yes | 20 |
| 116 | pass | 1.94 | 1.81 | accepted | yes | 21 |
| 117 | pass | 2.72 | 2.37 | accepted | yes | 22 |
| 118 | pass | 4.65 | 2.98 | accepted | yes | 23 |
| 119 | pass | 3.27 | 2.52 | accepted | yes | 23 |
| 120 | pass | 3.45 | 2.14 | accepted | yes | 23 |
| 121 | pass | 2.15 | 2.74 | accepted | yes | 23 |
| 122 | pass | 3.51 | 2.23 | accepted | yes | 23 |
| 123 | pass | 2.19 | 1.56 | accepted | yes | 23 |
| 124 | pass | 4.66 | 2.11 | accepted | yes | 23 |
| 125 | pass | 2.46 | 1.98 | accepted | yes | 23 |
| 126 | fail | 4.18 | 2.69 | rejected | no | 23 |
| 127 | pass | 1.83 | 2.01 | accepted | yes | 23 |
| 128 | pass | 3.2 | 2.95 | accepted | yes | 23 |
| 129 | pass | 5.06 | 3.33 | rejected | yes | 23 |
| 130 | pass | 5.06 | 3.33 | rejected | yes | 23 |
| 131 | pass | 4.29 | 3.19 | rejected | yes | 23 |
| 132 | pass | 2.7 | 2.62 | accepted | yes | 23 |
| 133 | pass | 3.6 | 2.36 | accepted | yes | 23 |
| 134 | pass | 2.98 | 2.26 | accepted | yes | 23 |
| 135 | pass | 4.31 | 2.25 | accepted | yes | 23 |
| 136 | pass | 3 | 2.74 | accepted | yes | 23 |
| 137 | pass | 4.37 | 2.47 | accepted | yes | 24 |
| 138 | pass | 4.51 | 2.78 | accepted | yes | 24 |
| 139 | pass | 2.39 | 1.59 | accepted | yes | 24 |
| 140 | pass | 2.02 | 2.25 | accepted | yes | 24 |
| 141 | fail | 1.98 | 1.65 | rejected | no | 24 |
| 142 | fail | 5.18 | 2.42 | rejected | no | 24 |
| 143 | pass | 4.45 | 3.77 | rejected | no | 25 |
| 144 | fail | 4.42 | 2.14 | rejected | no | 25 |
| 145 | pass | 3.11 | 2.42 | accepted | yes | 25 |
| 146 | pass | 2.84 | 1.7 | accepted | yes | 25 |
| 147 | pass | 4.32 | 3.15 | rejected | no | 25 |
| 148 | pass | 4.76 | 3.88 | rejected | no | 25 |
| 149 | pass | 3.03 | 2.34 | accepted | yes | 25 |
| 150 | pass | 2.67 | 1.43 | accepted | yes | 25 |
| 151 | fail | 5.36 | 2.4 | rejected | yes | 26 |
| 152 | pass | 2.94 | 1.91 | accepted | yes | 26 |
| 153 | fail | 4.67 | 2.88 | rejected | yes | 26 |
| 154 | pass | 4.59 | 3.89 | rejected | yes | 26 |
| 155 | pass | 2.95 | 2.73 | accepted | yes | 26 |
| 156 | pass | 2.34 | 2.03 | accepted | yes | 26 |
| 157 | pass | 3.43 | 1.69 | accepted | yes | 26 |
| 158 | pass | 5.11 | 2.59 | accepted | yes | 26 |
| 159 | pass | 1.43 | 1.43 | accepted | yes | 26 |
| 160 | pass | 2.44 | 2.05 | accepted | yes | 27 |
| 161 | pass | 2.69 | 1.93 | accepted | yes | 27 |
| 162 | pass | 2.75 | 3.28 | accepted | yes | 28 |
| 163 | pass | 1.76 | 1.39 | accepted | yes | 28 |
| 164 | pass | 4.44 | 2.89 | accepted | yes | 28 |
| 165 | pass | 2.98 | 2.73 | accepted | yes | 29 |
| 166 | pass | 3.46 | 2.82 | accepted | yes | 29 |
| 167 | pass | 3.65 | 1.71 | accepted | yes | 29 |
| 168 | pass | 3.3 | 2.45 | accepted | yes | 29 |
| 169 | pass | 2.16 | 1.78 | accepted | yes | 29 |
| 170 | pass | 3.5 | 1.6 | accepted | yes | 29 |
| 171 | pass | 4.12 | 2.11 | accepted | yes | 29 |
| 172 | pass | 2.58 | 2.09 | accepted | yes | 29 |
| 173 | pass | 2.9 | 2.35 | accepted | yes | 29 |
| 174 | pass | 3.43 | 2.42 | accepted | yes | 29 |
| 175 | pass | 2.65 | 2.51 | accepted | yes | 29 |
| 176 | pass | 2.38 | 1.94 | accepted | yes | 29 |
| 177 | pass | 3.67 | 2.64 | accepted | yes | 29 |
| 178 | pass | 2.58 | 2.38 | accepted | yes | 29 |
| 179 | pass | 2.56 | 2.17 | accepted | yes | 29 |
| 180 | pass | 1.81 | 1.97 | accepted | yes | 30 |
| 181 | pass | 4.38 | 2.24 | accepted | yes | 31 |
| 182 | pass | 4.06 | 2.6 | accepted | yes | 31 |
| 183 | pass | 2.56 | 2.19 | accepted | yes | 31 |
| 184 | pass | 2.22 | 2.01 | accepted | yes | 31 |
| 185 | fail | 4.31 | 2.51 | rejected | yes | 31 |
| 186 | pass | 4.96 | 2.68 | accepted | yes | 31 |
| 187 | pass | 4.56 | 2.78 | accepted | yes | 31 |
| 188 | pass | 3.48 | 2.47 | accepted | yes | 32 |
| 189 | pass | 2.62 | 2.25 | accepted | yes | 32 |
| 190 | pass | 2.58 | 1.73 | accepted | yes | 32 |
| 191 | pass | 2.78 | 2.73 | accepted | yes | 32 |
| 192 | pass | 2.93 | 1.63 | accepted | yes | 32 |
| 193 | pass | 3.92 | 2.4 | accepted | yes | 32 |
| 194 | pass | 4.05 | 2.84 | accepted | yes | 32 |
| 195 | pass | 3.63 | 2.08 | accepted | yes | 32 |
| 196 | pass | 2.81 | 2.31 | accepted | yes | 32 |
| 197 | pass | 4.17 | 2.55 | accepted | yes | 32 |
| 198 | fail | 1.55 | 2.15 | rejected | no | 33 |
| 199 | pass | 3.45 | 2.01 | accepted | yes | 33 |
| 200 | pass | 3.51 | 1.68 | accepted | yes | 33 |
| 201 | pass | 3.65 | 2.95 | accepted | yes | 33 |
| 202 | pass | 3.22 | 2.89 | accepted | yes | 33 |
| 203 | pass | 3.53 | 2.09 | accepted | yes | 33 |
| 204 | pass | 2.33 | 1.72 | accepted | yes | 33 |
| 205 | pass | 2.99 | 2.24 | accepted | yes | 33 |
| 206 | pass | 3.51 | 2.57 | accepted | yes | 33 |
| 207 | fail | 5.22 | 2.8 | rejected | yes | 33 |
| 208 | pass | 1.62 | 1.64 | accepted | yes | 33 |
| 209 | pass | 4.5 | 3.93 | rejected | no | 33 |
| 210 | pass | 4.63 | 3.45 | rejected | no | 33 |
| 211 | pass | 4.91 | 3.49 | rejected | no | 33 |
| 212 | pass | 6.18 | 3.47 | rejected | no | 33 |
| 213 | pass | 4.88 | 3.42 | rejected | no | 33 |
| 214 | pass | 3.6 | 2.6 | accepted | yes | 33 |
| 215 | pass | 3.87 | 2.93 | accepted | yes | 33 |
| 216 | pass | 3.08 | 2.73 | accepted | yes | 33 |
| 217 | pass | 3.21 | 2.21 | accepted | yes | 34 |
| 218 | pass | 3.5 | 2.36 | accepted | yes | 34 |
| 219 | pass | 3.25 | 2.06 | accepted | yes | 34 |
| 220 | pass | 3.92 | 2.02 | accepted | yes | 34 |
| 221 | pass | 2.75 | 1.84 | accepted | yes | 34 |
| 222 | pass | 2.83 | 1.4 | accepted | yes | 34 |
| 223 | fail | 4.28 | 2.64 | rejected | yes | 34 |
| 224 | pass | 5.15 | 2.55 | accepted | yes | 34 |
| 225 | pass | 3.98 | 2.08 | accepted | yes | 34 |
| 226 | pass | 1.6 | 2.11 | accepted | yes | 34 |
| 227 | pass | 1.67 | 1.54 | accepted | yes | 34 |
| 228 | pass | 1.78 | 1.76 | accepted | yes | 34 |
| 229 | pass | 3.63 | 2.94 | accepted | yes | 34 |
| 230 | pass | 3.6 | 2.95 | accepted | yes | 34 |
| 231 | pass | 3.4 | 2.43 | accepted | yes | 34 |
| 232 | pass | 2.98 | 2.88 | accepted | yes | 34 |
| 233 | pass | 3.58 | 2.31 | accepted | yes | 35 |

**References of the 233 compounds**

1. Raza S, Fransson L, Hult K: **Enantioselectivity in *Candida antarctica* lipase B: a molecular dynamics study.** *Protein Science* 2001, **10(2)**:329-338
2. Zhao TT, Zhang LJ, Gao J, Quan XJ: **Research on the synthesis of ester of lactic acid under inhibition of both substrates.** *Chinese Journal of Bioprocess Engineering* 2006, **4(3)**:51-55
3. Léonard V, Fransson L, Lamare S, Hult K, Graber M: **A water molecule in the stereospecificity pocket of *Candida antarctica* lipase B enhances enantioselectivity towards pentan-2-ol.** *Chembiochem* 2007, **8(6)**:662-667
4. Irimie FD, Tosa M, Paizs C, Majdik C, Moldovan P: **Biocatalytic synthesis of some novel (10-alkyl-10H-phenothiazine-3-yl)methyl acetates mediated by Lipase B from *Candida antarctica*.** *Roum. Biotechnol. Lett* 2001, **6(1)**:55-62
5. Conde S, Lopez SP, Martnez A: ***Candida antarctica* lipase B catalysed amidation of pyroglutamic acid derivatives.** *J. Mol.* *Catal. B: Enzym* 1999, **7(5)**:299-306
6. Orrenius C, Ohrner N, Rotticci D, Mattson A, Hult K, Norin T: ***Candida antarctica* Lipase B catalysed kinetic resolutions: substrate structure requirements for the preparation of enantiomerically enriched secondary alcanols***.* *Tetrahedron: Asymmetry* 1995, **6(5)**:1217-1220
7. Vargas RA, Diosa JE, Danieli B, Lesma G., Luisetti M, Riva S: ***Candida antarctica* Lipase B catalyzes the regioselective esterification of ecdysteroids at the C-2 OH.** *Tetrahedron* 1997, **53(16)**: 5855-5862
8. Hansen TV, Waagen V, Partali V, Anthonsen HW, Anthonsen T: **Co-solvent enhancement of enantioselectivity in lipase-catalysed hydrolysis of racemic esters. a process for production of homochiral C-3 building blocks using Lipase B from *Candida antarctica*.** *Tetrahedron: Asymmetry* 1995, **6(2)**:499-504
9. Rute ML, Menno JS, Giacomo C, Fred VR, Francesco S, Roger AS: **Dissolution of *Candida antarctica* lipase B in ionic liquids: effects on structure and activity.** *Green Chem* 2004, **6**:483 – 487
10. Gotor FV, Busto E, Gotor V: ***Candida antarctica* Lipase B: An Ideal Biocatalyst for the Preparation of Nitrogenated Organic Compounds.** *Advanced synthesis & catalysis* 2006, **348(7-8)**:797-812
11. Lou WY, Zong MH, Liu YY, Wang JF: **Efficient enantioselective hydrolysis of D,L-phenylglycine methyl ester catalyzed by immobilized *Candida antarctica* lipase B in ionic liquid containing systems.** *J. Biotechnol* 2006, **25(1)**:64-74
12. Jacobsen EE, Van HE, Moen AR, Vazquez PLC, Anthonsen T: **Enhanced selectivity in Novozym 435 catalyzed kinetic resolution of secondary alcohols and butanoates caused by the (R)-alcohols.** *Tetrahedron letters* 2003, 44(46):8453-8455
13. Skupinska KA, McEachern EJ, Baird IR, Skerlj RT, Bridger GJ: **Enzymatic resolution of bicyclic 1-heteroarylamines using *Candida antarctica* lipase B.** *J. Org. Chem* 2003, **68(9)**:3546-3551
14. Patel RN, Banerjee A, Nanduri V, Goswami A, Comezogluf T: **Enzymatic resolution of racemic secondary alcohols by lipase B from *Candida antarctica*.** *Journal of the American Oil Chemists' Society* 2000, **77(10)**:1015-1019
15. Vieira TO, Ferraz HMC, Andrade LH, Porto ALM: **Highly enantioselective enzymatic resolution of cis-fused octalols mediated by *Candida antarctica* lipase.** *Tetrahedron : Asymmetry* 2006, **17(13)**:1990-1994
16. Ottosson J, Hult K: **Influence of acyl chain length on the enantioselectivity of *Candida antarctica* lipase B and its thermodynamic components in kinetic resolution of sec-alcohols.** *J. Mol.* *Catal. B: Enzym* 2001, **11(4)**:1025-1028
17. Arroyo M, Sinisterra JV: **Influence of chiral carvones on selectivity of pure lipase-b from *Candida antarctica*.** *Biotechnology Letters* 1995, **17(5)**:525-530
18. Hoff BH, Ljones L, Ronstad A, Anthonsen T: **Influence of substituents on enantiomeric ratio in transesterification of racemic C-3 synthons using lipase B from *Candida antarctica*.** *J. Mol.* *Catal. B: Enzym* 2000, **8(1)**:51-60
19. Kourist R, Gonzalez SJ, Liz R, Rebolledo F: **Kinetic resolution of 1-biaryl- and 1-(pyridylphenyl)alkan-1-ols catalysed by the lipase B from *Candida antarctica*.** *Advanced synthesis & catalysis* 2005, 347(5):695-702
20. Javier GS, Vicente G, Francisca R: **Kinetic resolution of (±)-trans- and (±)-cis-2-phenylcyclopentanamine by CALB-catalyzed aminolysis of esters: the key role of the leaving group.** *Tetrahedron: Asymmetry* 2004, 15(3):481-488
21. Barz M, Herdtweck E, Thiel WR: **Kinetic resolution of trans-2-(1-pyrazolyl)cyclohexan-1-ol catalyzed by Lipase B from *Candida antarctica*.** *Tetrahedron: Asymmetry* 1996, **7(6)**:1717-1722(6)
22. Eduardo GU, Francisca R, Vicente G: **Kinetic resolution of (±)-1-phenylbutan-1-ol by means of CALB-catalyzed aminolyses: a study on the role of the amine in the alcohol resolution.** *Advanced Synthesis & Catalysis* 2001, **343(6-7)**:646 – 654
23. Agnes DC, Milou LC, Kouwijzer E, Peter DJG, Richard MK, Ben LF: **Kinetic resolutions and enantioselective transformations of 5-(acyloxy)pyrrolinones Using *Candida antarctica* Lipas*e* B: synthetic and structural aspects.** *J. Org. Chem* 1999, **64**:9529-9537
24. Ohrner N, Orrenius C, Mattson A, Norin T, Hult K: **Kinetic resolutions of amine and thiol analogues of secondary alcohols catalyzed by the *Candida antarctica* lipase B.** *Enzyme and Microbial Technology* 1996, **19(5)**:328-331
25. Brunet C, Zarevucka M, Wimmer Z, Legoy MD: **Lipase B from *Candida antarctica* catalyses enantioselective transesterification of 2-(4-methoxybenzyl)-1-cyclohexanols and 2-(4-methoxybenzyl)-1-cyclopentanols.** *Biotechnology Letters* 1999, **21(7)**:605-610
26. Yeon SL, Joo HH, Nan YJ, Won K, But TK: **Highly enantioselective acylation of rac-alkyl lactates using *Candida antarctica* lipase B.** *Organic Process Research & Development* 2004, **8(6)**:948-951
27. Palomo JM, Fernandez LG, Mateo C, Fuentes M, Fernandez LR, Guisan JM: **Modulation of the enantioselectivity of *Candida antarctica* B lipase via conformational engineering. Kinetic resolution of (+/-)-
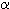
-hydroxy-phenylacetic acid derivatives.** *Tetrahedron: Asymmetry* 2002, **13(12)**:1337-1345
28. Fuglseth E, Anthonsen T, Hoff BH: **New chiral building blocks from acetovanillone using lipase A and B from *Candida antarctica*.** *Tetrahedron: Asymmetry* 2006, **17(8)**:1290-1295
29. Xu D, Li Z, Ma S: **Novozym-435-catalyzed enzymatic separation of racemic propargylic alcohols. A facile route to optically active terminal aryl propargylic alcohols.** *Tetrahedron Letters* 2003, **44(33)**:6343-6346
30. Li XF, Zong MH, Yang RD: **Novozym 435-catalyzed regioselective acylation of 1-β-D-arabinofuranosylcytosine in a co-solvent mixture of pyridine and isopropyl ether.** *J. Mol.* *Catal. B: Enzym* 2006, **38(1)**:48-53
31. Palomo JM, Mateo C, Fernandez LG, Solares LF, Diaz M, Sanchez VM, Bayod M, Gotor V, Guisan JM, Fernandez LR: **Resolution of (+/-)-5-substituted-6-(5-chloropyridin-2-yl)-7-oxo-5,6-dihydropyrrolo[3,4b]p yrazine derivatives-precursors of (S)-(+)-Zopiclone, catalyzed by immobilized *Candida antarctica* B lipase in aqueous media.** *Tetrahedron: Asymmetry* 2003, **14(4)**:429-438
32. Gedey S, Liljeblad A, Lázár L, Fülöp F, Kanerva LT: **Structural effects on chemo- and enantioselectivity of *Candida antarctica* lipase B - Resolution of
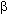
-amino esters.** *Canadian Journal of Chemistry* 2002, **80(6)**:565-570
33. Otto RT, Scheib H, Bornscheuer UT, Pleiss J, Syldatk C, Schmid RD: **Substrate specificity of lipase B from *Candida antarctica* in the synthesis of arylaliphatic glycolipids.** *J. Mol.* *Catal. B: Enzym* 2000, **8(4)**:201-211
34. Torres GA, Castilloe OE, Lopez MA: **The amidase activity of *Candida antarctica* lipase B is dependent on specific structural features of the substrates.** *J. Mol.* *Catal. B: Enzym* 2006, **41(3-4)**:136-140
35. Orrenius C, Nortin T, Hult K, Carrea G: **The *Candida antarctica* lipase B catalysed kinetic resolution of seudenol in non-aqueous media of controlled water activity.** *Tetrahedron : Asymmetry* 1995, **6(2)**:3023-3030
