# Supplementary material for: Structure-based substrate screening for an enzyme
Source: BMC Bioinformatics. 2009 Aug 21;10:257. doi: 10.1186/1471-2105-10-257 (PMC2745390; doi:10.1186/1471-2105-10-257)
Supplement: Additional file 3 — Docking N-hexylphosphonate ethyl ester (HEE) in 1TCA and 1LBS. This part compares the results of the self-docking experiment using 1TCA and 1LBS as the target structure in detail. [file 1471-2105-10-257-S3.doc]

## Docking N-hexylphosphonate ethyl ester (HEE) in to 1TCA and 1LBS using two different initial binding conformation

The N-hexylphosphonate ethyl ester (HEE) were extracted from the crystal structure of 1LBS and directly used for docking. Two initial binding conformations (figure s1) were used. One is the exact the binding conformation that was found in the transition state ligand bound crystal structure (1LBS), the other is the one that the positions of the n-hexyl group was exchanged with that of “-OCH_2_CH_3_” group (the positions of some other atoms were changed as well). The ligand (HEE) was docked into the same binding site of two different crystal structure, 1TCA (Ligand free crystal structure) and 1LBS (transition state analog crystal structure) respectively. Docking protocols and all settings were the same as those of “Docking engine – Affinity” section of the manuscript, and Affinity would produce four binding poses between enzyme and HEE.


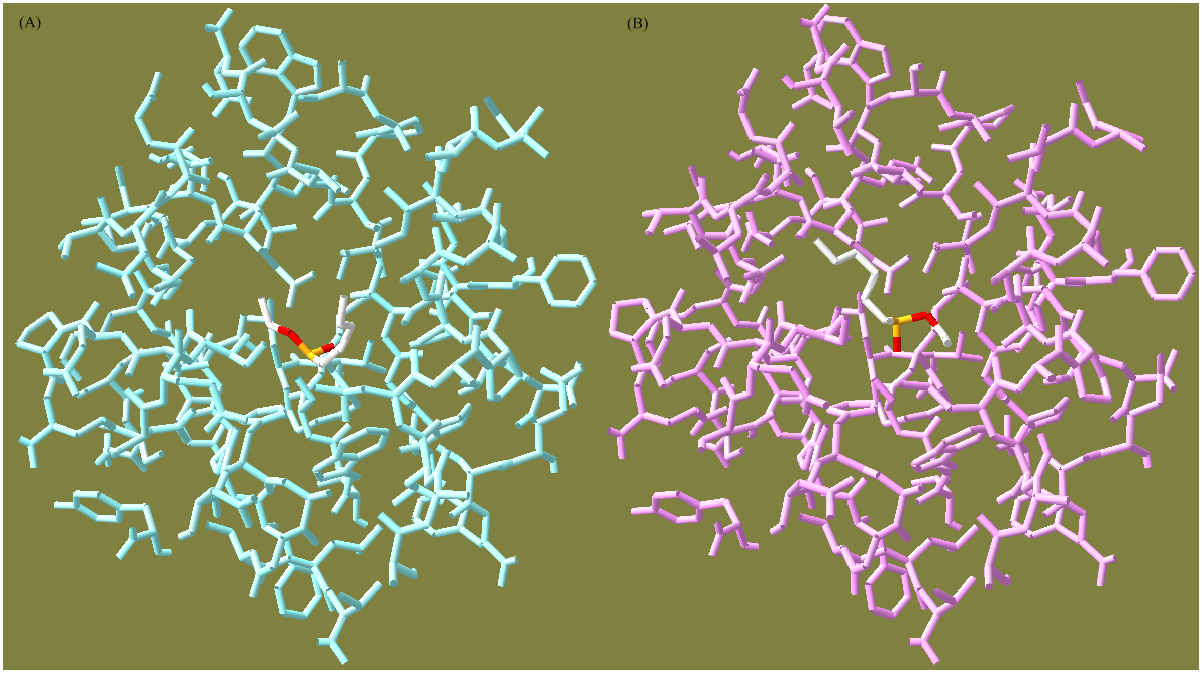


Figure s1 Initial binding orientation of the HEE. (A) experimentally determined binding conformation; (B) conformation in which the positions of n-hexyl group and that of “-OCH_2_CH_3_” group exchanged.

For the sake of convenience, conformations in figure s1A and figure s1B was renamed “conformation A” and “conformation B” separately.

1. **“Conformation A” as the initial binding posture**

“Conformation A” is the exact ligand binding conformation that was found in the ligand bound crystal structure (1LBS). Using it as the initial binding posture, however, could bias the self-docking experiment (the reason was explained in the nest paragraph). As table s1 showed, either of the structure 1TCA or 1LBS could produce only one final binding pose although the maximum number of the accepted binding pose were set four. And both of the accepted binding poses were similar to the experimentally determined ligand conformation (figure s2 and figure s3). This observation provided direct evidence to our assumptions about the self-docking bias caused by the initial binding conformation.

Table S1 Binding poses of Affinity using“Conformation A” as the initial binding posture

| Structure for docking | Potential energy (kcal/mol) | RMSD (Å) |
| --- | --- | --- |
| 1TCA | -19.47 | 1.12 |
| 1LBS | -8.63 | 0.50 |


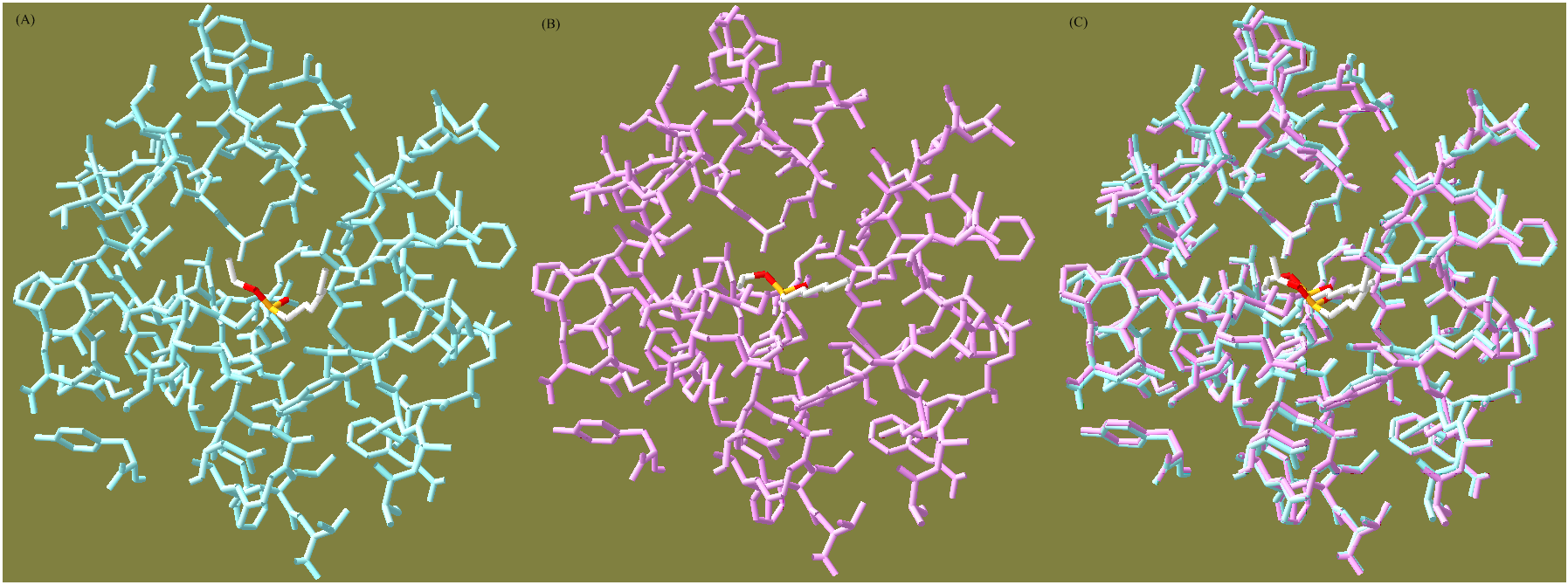


Figure s2 Docking HEE into 1TCA using “conformation A” as the initial binding conformation. (A) Original binding orientation of HEE in 1LBS. (B) Binding orientation of HEE when it was docked into 1TCA. (C) Superimposition of orientation of A and B.


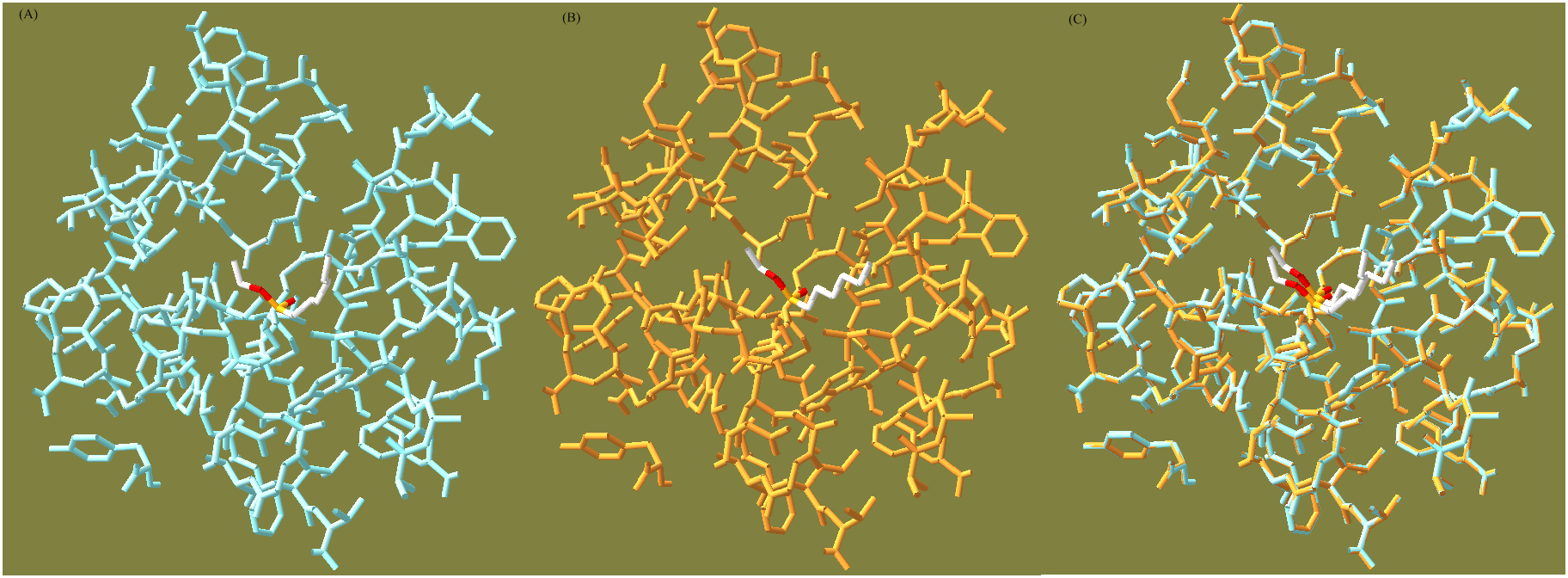


Figure s3 Docking HEE into 1LBS using “conformation A” as the initial binding conformation. (A) Original binding orientation of HEE in 1LBS. (B) Binding orientation of HEE when it was docked into 1LBS. (C) Superimposition of orientation of A and B.

The docking bias of “conformation A” was probably caused by the docking engine-Affinity. According to its docking scheme, initial enzyme-substrate complex would be first minimized to obtain the starting structure. After that, Affinity moved the ligand by random combination of translation, rotation, and torsional changes to create other new binding conformation. Then Affinity subsequently checked the energy of the resulting randomly moved structure. If it was within the energy tolerance parameter (1000 Kcal/mol) of the previous minimized structure, it was considered to have passed the first step and the structure was then subjected to energy minimization. The final minimized structure was accepted or rejected based on the energy criterion (Metropolis standard) and its similarity to structures found before. if the experimentally determined ligand conformation (“conformation A”) was used, few new energy-minimized binding poses could pass the energy check because energy of “conformation A” was already the lowest or close to the lowest after the initial minimization by Affinity. In this sense, Affinity would probably fail most of the new pose searching and accept the minimized initial “conformation A” as the final binding pose.

1. **“Conformation B” as the initial binding posture**

In order to avoid the bias caused by the initial binding pose, we built “conformation B” as the initial binding orientations. Its conformation and orientation were totally different from the experimentally determined “conformation A”. So its energy was no longer the lowest. By randomly place the ligand in the binding pocket, Affinity could get over any energy barrier on the potential energy surface and produce any binding poses. However, the random placing in some cases could potentially lead to very severe divergences in the coulombic and vdW energies. New binding poses would be rejected and thus not be subject to the minimization step because of its extraordinarily large energy than previous structure. This limits the searching space again. So the coulombic term and vdW term needs to be scaled down to tolerate more new binding poses. Otherwise, the final binding poses would still be similar to the initial binding conformation and lie in a local minimum of the potential energy surface. If the scale factor is too large (e.g. 10^-2^), searching space of Affinity is still limited due to the large coulombic and vdW energies. However, If the scale factor is too small (e.g. 10^-15^), searching space of Affinity would become too large, and the efficiency of finding possible binding poses become too low due to the long searching time. Since docking protocols and all settings were the same as that of “Docking engine – Affinity” section of the manuscript, the scale factor of the coulombic term and vdW term were 10^-7^.

The self-docking result showed both structures (1TCA and 1LBS) could produce four final binding poses (table s2). As table s2 displayed, RMSD between the docked ligand and the ligand found in crystal structure were 1.35 Å and 1.54 Å for 1TCA and 1LBS respectively. This suggested that 1TCA could reproduce the experimentally determined ligand conformation better than 1LBS could to (fig.s4 and fig.s5). Besides, energy of the binding pose of 1TCA was much lower. This indicated that using 1TCA as the target structure for docking would probably produce more stable binding pose. The higher resolution of the crystal structure1TCA (R=1.55 Å) may have influence on the final energy of the enzyme-ligand complex. In this sense, we thought 1TCA outperformed 1LBS in the self-docking experiment and we preferably used 1TCA as the crystal structure for the substrate screening system.

Table s2 Binding poses of Affinity using“Conformation B” as the initial binding posture

| Structure for docking | Final binding poses | Energy (kcal/mol) | RMSD (Å) |
| --- | --- | --- | --- |
| 1TCA | 1 | -20.83 | 1.35 |
|  | 2 | -19.62 | 1.42 |
|  | 3 | -19.17 | n.d. |
|  | 4 | -18.64 | n.d. |
|  |  |  |  |
| 1LBS | 1 | -7.60 | 1.54 |
|  | 2 | -6.09 | n.d. |
|  | 3 | -4.96 | 1.57 |
|  | 4 | -4.76 | 1.69 |

“n.d.” suggested that ligand in this pose displayed a totally different binding orientation from what was found in 1LBS.


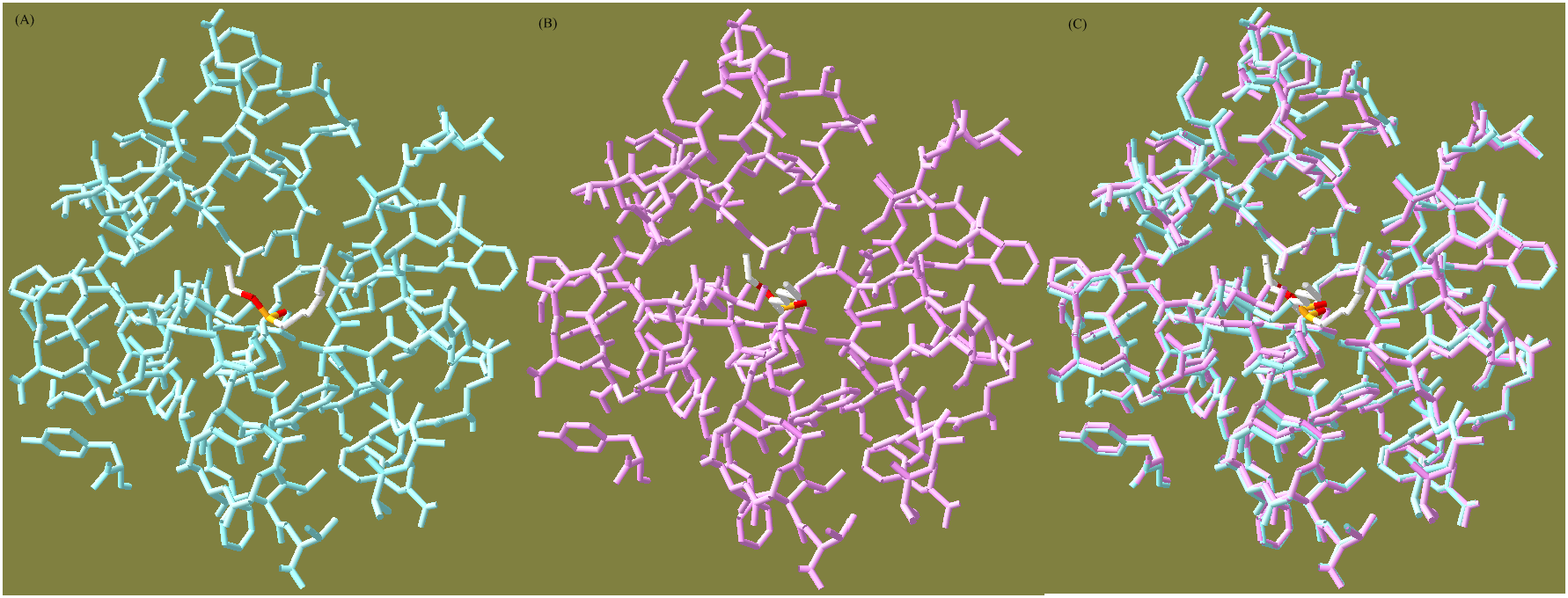


Figure s4 Final binding pose 1 when 1TCA was used the crystal structure. (A) Original binding orientation of HEE in 1LBS. (B) binding pose 1. (C) Superimposition of orientation of A and B.


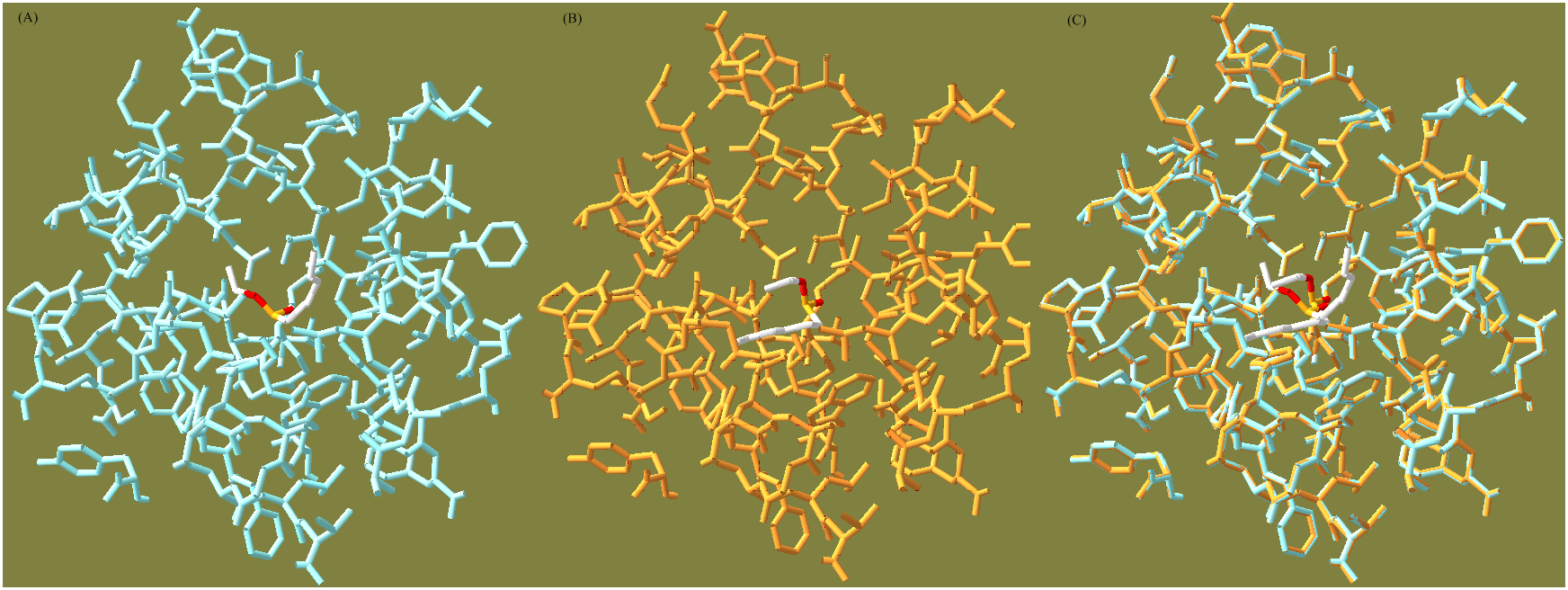


Figure s5 Final binding pose 1 when 1LBS was used the crystal structure. (A) Original binding orientation of HEE in 1LBS. (B) binding pose 1. (C) Superimposition of orientation of A and B.

The rest binding poses of both structures were also listed in the following figures.

**Binding pose 2 of the 1TCA**


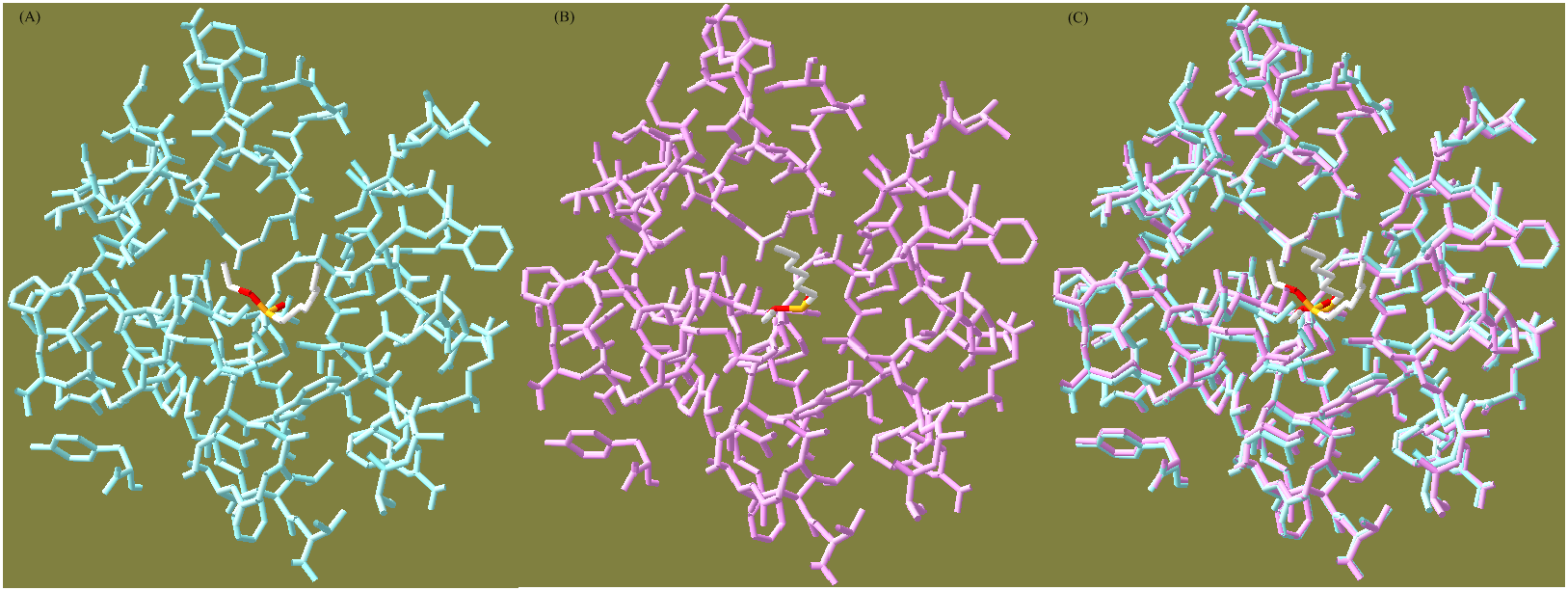


Figure s6 Final binding pose 2 when 1TCA was used the crystal structure. (A) Original binding orientation of HEE in 1LBS. (B) binding pose 2. (C) Superimposition of orientation of A and B.

**Binding pose 3 of the 1TCA**


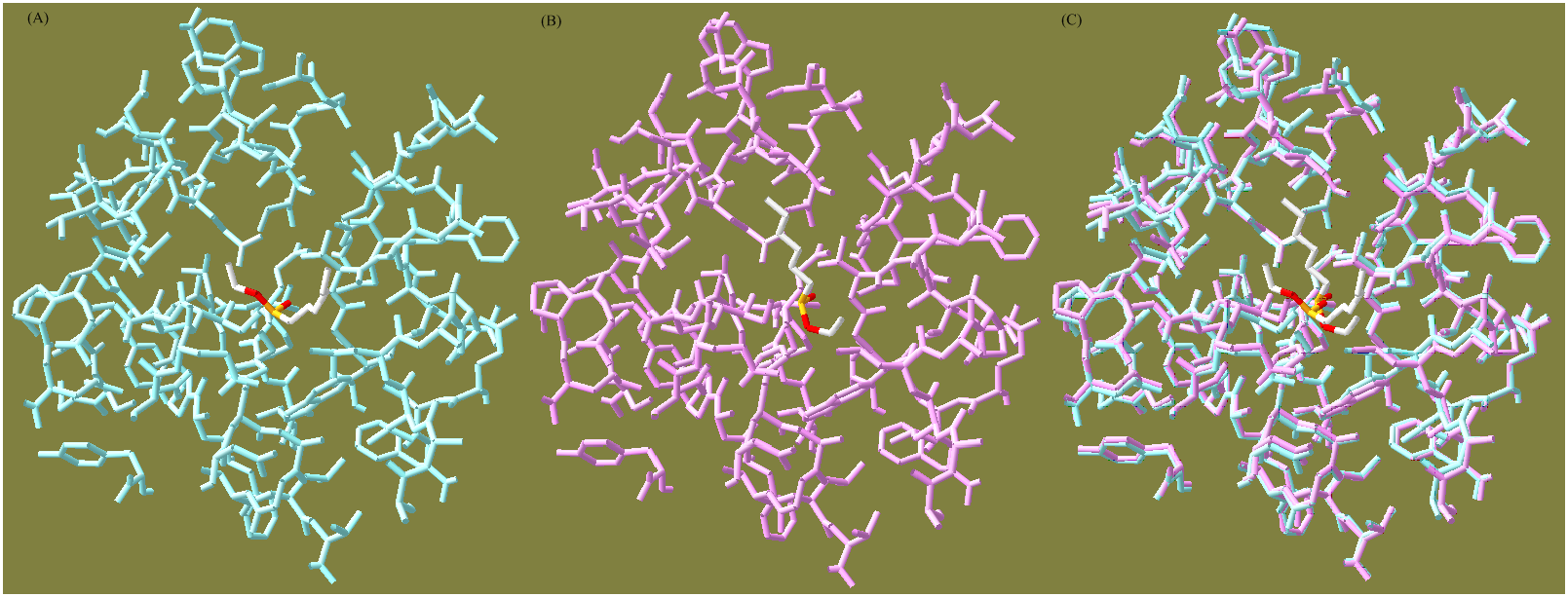


Figure s7 Final binding pose 3 when 1TCA was used the crystal structure. (A) Original binding orientation of HEE in 1LBS. (B) binding pose 3. (C) Superimposition of orientation of A and B.

**Binding pose 4 of the 1TCA**


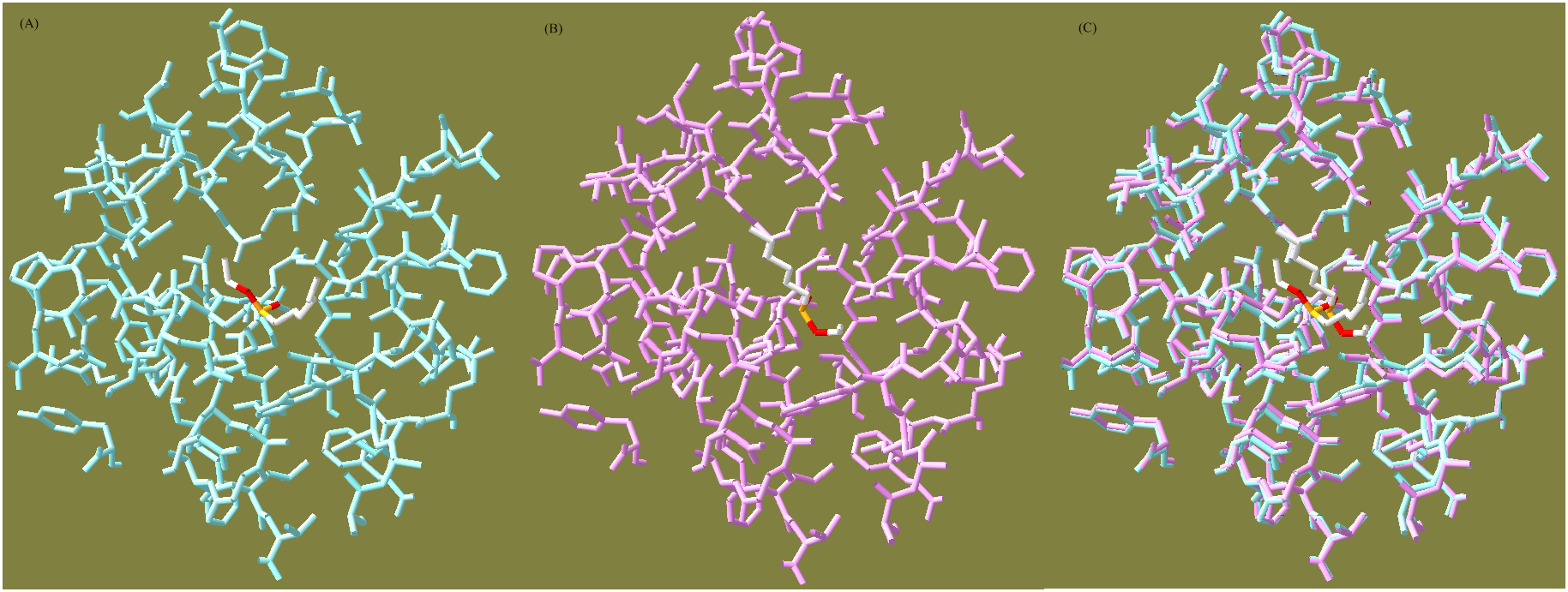


Figure s8 Final binding pose 4 when 1TCA was used the crystal structure. (A) Original binding orientation of HEE in 1LBS. (B) binding pose 4. (C) Superimposition of orientation of A and B.

**Binding pose 2 of the 1LBS**


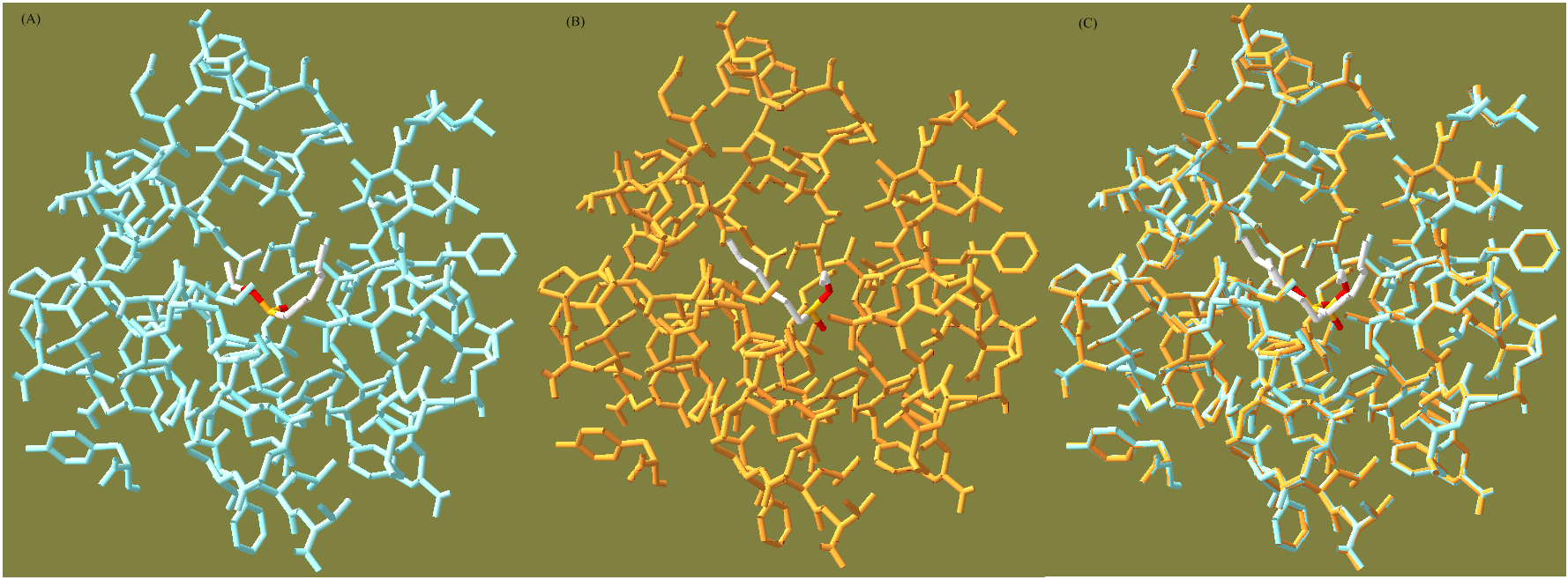


Figure s9 Final binding pose 2 when 1LBS was used the crystal structure. (A) Original binding orientation of HEE in 1LBS. (B) binding pose 2. (C) Superimposition of orientation of A and B.

**Binding pose 3 of the 1LBS**


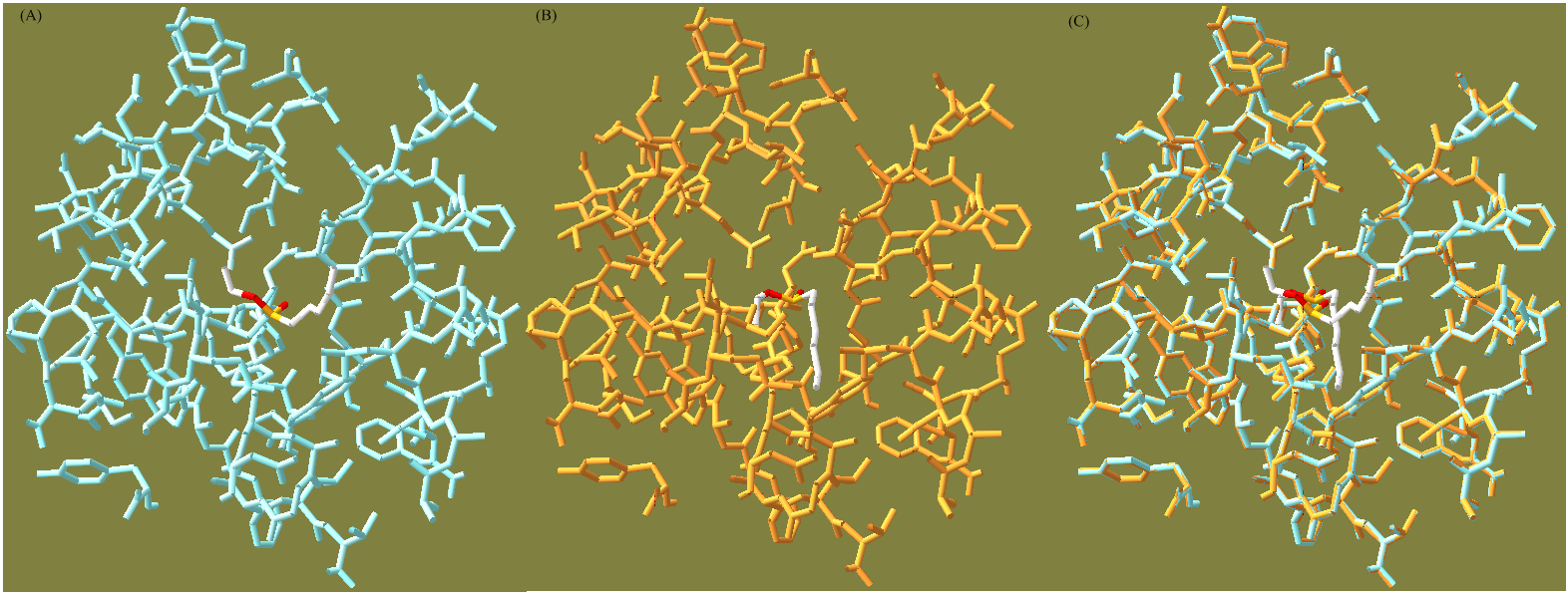


Figure s10 Final binding pose 3 when 1LBS was used the crystal structure. (A) Original binding orientation of HEE in 1LBS. (B) binding pose 3. (C) Superimposition of orientation of A and B.

**Binding pose 4 of the 1LBS**


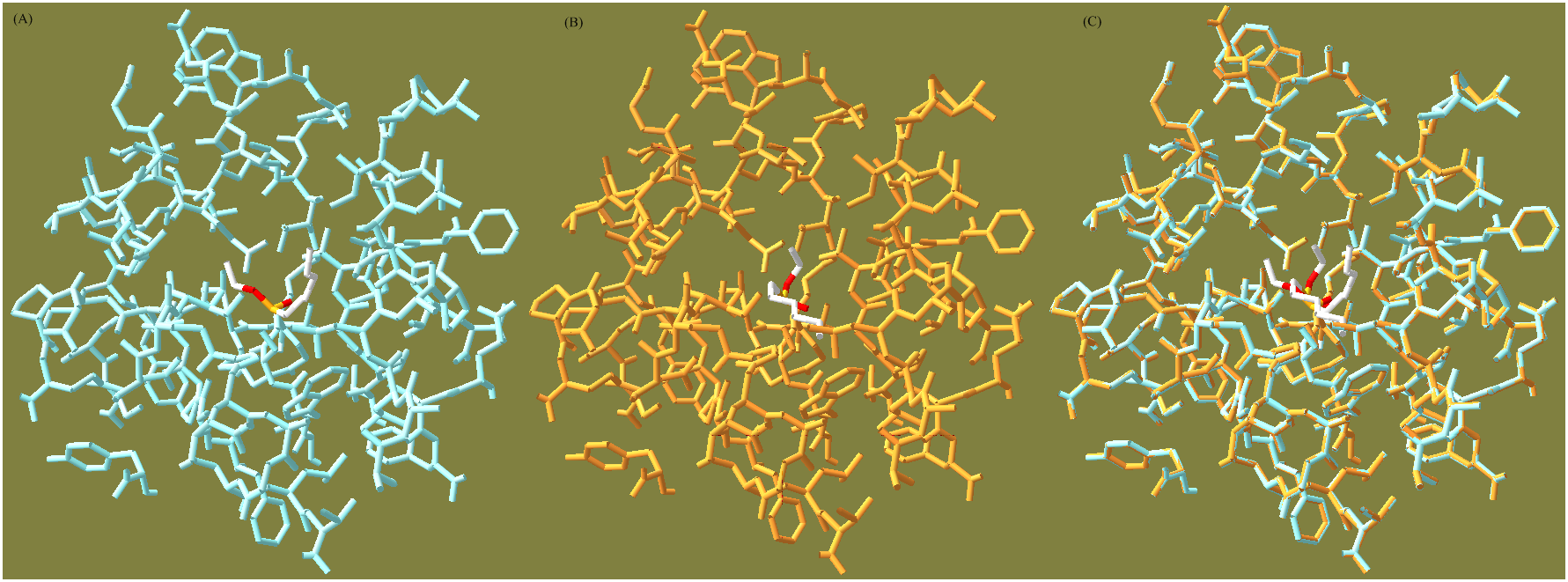


Figure s11 Final binding pose 4 when 1LBS was used the crystal structure. (A) Original binding orientation of HEE in 1LBS. (B) binding pose 4. (C) Superimposition of orientation of A and B.
