# Supplementary material for: Structure-based substrate screening for an enzyme
Source: BMC Bioinformatics. 2009 Aug 21;10:257. doi: 10.1186/1471-2105-10-257 (PMC2745390; doi:10.1186/1471-2105-10-257)
Supplement: Additional file 4 — MD simulation of the transition state of CALB-substrate complex. This part displays a detailed description of the preparation of transition state of enzyme-substrate complex and the following MD simulation results. Table S1 shows the result of MD simulation. [file 1471-2105-10-257-S4.doc]

**Construction of the transition state form of the enzyme-compound complex and its following MD simulation**

The transition state form of the enzyme-compound complex was manually modeled on the basis of crystal structures of CALB with a phosphonate inhibitor, that was, a transition state analog (PDB code 1LBS) and an ester with a sufficiently long acyl chain on which to base the conformations of the acyl moieties in the models (PDB code 1LBT).After a few hundreds of minimization of the enzyme-substrate transition state, the system run through an MD warm-up phase to a temperature of 300K in a series of 10 steps at 30K intervals, where the simulated duration of each interval was 1ps. Thereafter, a 150-ps MD simulation was performed at 300 K for each system. A sample structure was extracted at every 0.1ps from each simulation. Investigative analyses were performed on the last 25 ps of each simulation, yielding an ensemble of 250 nonminimized structures for each system. Average potential energies of the two different initial (as figure 7 of the manuscript showed) binding conformations for the same compound were calculated and then compared each other to determine a better final conformation. After that, the better conformation was compared with the conformation predicted by the proposed conformational rule to validate the “Conformational Check”. For the sake of convenience, conformation in figure 7A and figure 7B was named A and B, respectively.

**Result of MD simulation for verification of Conformational Check**

Enzyme-substrate complex whose potential energy was lower among the two initial binding conformations was thought as the more stable conformation, and its possibility of being the exact binding orientation when a real reaction happened was much larger. As table s1 showed, enzyme-substrate complexes which were predicted to be more stable by MD simulations were always in consistence with the conformation predicted by our “larger part of compound binds to larger part of enzyme” rule. This suggested that the proposed conformational rule was available and accurate in defining the binding conformation of substrate. So it was named as the “Conformational Check” and would be used as the first screening criterion in CASS.

Table S1 Results of the MD simulation

| Compound | Initial conformation | Average potential energy (Kcal/mol) | Standard Deviation of potential energy (Kcal/mol) | Conformation predicted by conformational rule | More stable conformation predicted by MD simulation |
| --- | --- | --- | --- | --- | --- |
| A | B | -34537.11 | 39.79 | A | A |
|  | A | -35385.78 | 45.12 |  |  |
| B | B | -34627.17 | 40.73 | B | B |
|  | A | -34573.04 | 41.03 |  |  |
| C | B | -34595.51 | 42.74 | B | B |
|  | A | -34526.08 | 40.75 |  |  |
| D | B | -34943.94 | 40.36 | A | A |
|  | A | -35099.23 | 39.69 |  |  |
| E | B | -34688.34 | 42.21 | B | B |
|  | A | -34647.13 | 43.52 |  |  |
| F | B | -34815.68 | 40.42 | A | A |
|  | A | -35234.29 | 45.75 |  |  |
| G | B | -35148.20 | 41.31 | B | B |
|  | A | -34413.38 | 45.32 |  |  |
| H | B | -34905.01 | 46.63 | A | A |
|  | A | -35026.93 | 41.15 |  |  |
